# Dual Quaternion Framework for Modeling of Spacecraft-Mounted Multibody Robotic Systems

**DOI:** 10.3389/frobt.2018.00128

**Published:** 2018-11-21

**Authors:** Alfredo Valverde, Panagiotis Tsiotras

**Affiliations:** Dynamics and Control Systems Laboratory, School of Aerospace Engineering, Georgia Institute of Technology, Atlanta, GA, United States

**Keywords:** multibody dynamics, dual quaternions, space operations, robotic servicing, Newton-Euler

## Abstract

This paper lays out a framework to model the kinematics and dynamics of a rigid spacecraft-mounted multibody robotic system. The framework is based on dual quaternion algebra, which combines rotational and translational information in a compact representation. Based on a Newton-Euler formulation, the proposed framework sets up a system of equations in which the dual accelerations of each of the bodies and the reaction wrenches at the joints are the unknowns. Five different joint types are considered in this framework via simple changes in certain mapping matrices that correspond to the joint variables. This differs from previous approaches that require the addition of extra terms that are joint-type dependent, and which decouple the rotational and translational dynamics.

## 1. Introduction

The interest to operate servicing spacecraft in space has led to wide-ranging research in academia, governmental agencies, and private companies. The space servicing market is growing, and with it, also the need for effective and easy-to-use tools to model the different phases of the mission. One tool of particular interest that has garnered attention for proximity operations, during which not only the attitude, but also the position of a spacecraft has to be precisely controlled, are dual quaternions, see for example Filipe and Tsiotras (2013a), Seo (2015), and Filipe et al. (2015). We add to this body of literature, having as a goal to provide an intuitive development of the multibody dynamics of a spacecraft-mounted manipulator system in dual quaternion algebra using a Newton-Euler approach. The aim is to provide a unified mathematical framework in which to model the different phases of a servicing mission.

### 1.1. Multibody dynamics for space applications

When it comes to mounting a robotic manipulator on a spacecraft, the development of the equations of motion is not as straightforward as ground-based robotic applications, due to the complex interaction between reaction forces that arise at the joints and the conservation of angular momentum. This is especially important for relatively small spacecraft with large manipulators, as the stationarity assumption of the base is not longer valid. In such scenarios (that become increasingly popular in practice) the combined base-manipulator motion has to be accounted for. In terms of prior work in spacecraft equipped with manipulators, we can mention Hooker (1970), who first derived the equations of motion for an n-body satellite. In his derivation, the reaction forces and torques at the joints are not explicit in the formulation and aims to expose the body axes so that it is convenient to incorporate control laws, internal forces and other disturbance forces into the model that would not be straightforward to introduce using a Lagrangian formulation. Hooker's approach is based on the addition of the independent equations of motion for each of the bodies to cancel the reaction forces, and the cancellation of reaction torques through a clever manipulation of the equations of motion. This leads to a system of equations where the unknowns are the angular acceleration of the base, and the generalized accelerations at the joints. Longman et al. (1987) proposed a model for the operation of a robotic arm mounted on the Space Shuttle when attitude control is enabled. The authors in Longman et al. (1987) developed a forward and inverse kinematic model based on an initial determination of where the center of mass of the system is. This allows for identification of where the satellite-body is in inertial space as a function of joint angles, enabling a custom-derived solution of the forward and inverse kinematics problem. The authors then provided an approach to extract the reaction forces and torques applied on the satellite base due to the robotic arm through the extension of results derived using a fixed-base approach. Umetani and Yoshida (1989) introduced the equations of motion for systems with revolute joints and an uncontrolled (not actively controlled) base. The authors introduce an innovative representation of the task-space motion of the end-effector using two Jacobians—one capturing the effect of the spacecraft's motion, and another one to capture the effect ofjoint rates. This framework, however, did not account for external forces or torques. Dubowsky et al. (1989) dealt with the problem of thruster, or joint actuator saturation as an integral part of path-planning for the manipulators. Their model consists of a nine-generalized-coordinate system, and they derive the equations of motion using the Lagrangian approach, involving a 9 × 9 × 9 tensor to compute the Coriolis-like term. Papadopoulos and Dubowsky (1991a) rewrite the equations of motion of the satellite-mounted robot arm, but this time include actuation of the satellite base, and embed them in a quasi-Lagrangian approach.

Papadopoulos and Dubowsky (1990, 1991b) succinctly describe the equations of motion for a robotic arm on a satellite under the assumption of zero initial angular momentum using the *Routhian* and a compact representation of the kinetic energy of the system. The authors proceed to argue that fixed-base and space-based manipulators can *almost* always be controlled using the same control algorithms, given the structural similarities between the corresponding model matrices. Xu and Shum (1991) developed a dynamical model for a robotic arm mounted on a satellite base in the absence of thruster jets. This implies that the motion of the system obeys the conservation of linear and angular momentum, a fundamental fact in their derivation. Walker and Weel (1991) provided the equations of motion for a six degree of freedom robotic arm on a satellite base. The method incorporates three reaction wheels and the equations are derived using a Lagrangian formulation. They eliminate the velocity of the satellite base from this formulation, given the constraint of no external forces on the system, without necessarily assuming that the initial momenta of the system are zero. Their formulation leads to a complicated system of equations that relies on the pre-computation of a significant amount of partial derivatives. Carignan and Akin (2000) proposed a recursive Newton-Euler algorithm that is easy to implement, intuitive, and has been well adopted by the engineering community. As an example, Dubanchet et al. (2015) hinges on this dynamics framework to implement *H*_∞_ control on a linearized version of the plant with the objective of designing a debris collection robotic manipulator in space. Stoneking (2007) uses a Newton-Euler approach which exposes the reaction forces of the system, solved for by a matrix inversion that also yields linear and angular accelerations. The same author proposes a decoupling of the equations for users not interested in the reaction forces at the joints. Furthermore, he provides a formulation for the case in which the joints are not given by a simple primitive (revolute or prismatic), such as is the case of a gimbaled joint. Bishop et al. (2014) used this method for path planning and control during rapid maneuvering of a robotic arm mounted on a spacecraft. Stoneking (2013) also proposed an approach based on Kane's equations of motion, in which the generalized coordinates appear as part of a minimal representation. In this case, extracting knowledge about the reaction forces and torques, which are particularly important during design phases, becomes a significantly more complicated task.

Jain (1991) and Rodriguez et al. (1991, 1992) provided a numerically efficient multibody dynamics framework based on spatial operator algebra. Featherstone and Orin (2000) and Featherstone (2008), provided generalizable algorithms to model multibody dynamics. In particular, in section 9.3 of Featherstone (2008) the author specializes his algorithm to free-floating bases. Saha (1999), Mohan and Saha (2007), and Saha et al. (2013) provided another numerical algorithm for recursive dynamics, which claims to be even more efficient than the one by Featherstone, and it relies on using projection matrices to eliminate reaction forces and torques. Software has also been developed to model general dynamical systems. For example, Moosavian and Papadopoulos (2004) describes SPACEMAPLE, a tool that uses an analytical formulation of the Lagrangian equations of motion. At Tohoku University in Japan, Yoshida (1999) and his research team developed the SpaceDyn toolbox. The toolbox uses a recursive Newton-Euler approach, method further explored by Carignan and Akin (2000). Other open source toolboxes available online include SPART by Virgili-Llop et al. (2017), developed specifically for spacecraft-mounted manipulators, and DART by Lee et al. (2018), which is aimed for general multibody systems, among many others. Commercial software packages also exist. Among these, SD/FAST by Sherman and Rosenthal (2001) is a commonly used software package for spacecraft modeling.

In this wide literature for dynamic modeling of spacecraft-mounted robotic manipulators, dual quaternions are mentioned and used only a handful of times. In particular, Dooley and McCarthy (1993) proposed using dual quaternions as generalized coordinates, and Brodsky and Shoham (1999) proposed a rigorous dual-number based methodology that resulted in a Lagrangian-like framework. Brodsky and Shoham did draw parallelisms with a Newton-Euler-type equation, but these were always projected onto the dual axes of motion for the cases concerning serial manipulators, obscuring any potential insight into the reaction forces and torques at the joints.

The lack of previous work using dual quaternions in a classical Newton-Euler framework to model serial manipulator systems on a spacecraft motivated the work of this paper. In particular, the highly generalizable dynamics framework presented herein aims exploits the versatility of dual quaternions to capture coupled rotational and translational dynamic quantities, and to capture joint kinematic constraints at both, the velocity and the acceleration levels. The framework is developed using dual quaternions, an extension of the well-known quaternions, a mathematical language that is familiar to the practitioner in the field of spacecraft dynamics and control. Additionally, the proposed framework consists of a non-recursive approach that solves a well-defined system of equations for a satellite with a tree-like architecture. By providing a simple-to-follow algorithm, the proposed work aims at increasing the accessibility of the uninitiated into the realm of multibody dynamics.

## 2. Mathematical preliminaries

### 2.1. Quaternions

The group of quaternions as defined by Hamilton in 1843 extends the well-known imaginary unit *j*, which satisfies the equation *j*^2^ = −1. This non-abelian group is defined by the presentation $Q_{8}≜\left\{-1,i,j,k:i^{2}=j^{2}=k^{2}=ijk=-1\right\}$. The algebra constructed from *Q*_8_ over the field of real numbers is the quaternion algebra, and it gives rise to the set ℍ. We define quaternions as $ℍ≜\left\{q=q_{0}+q_{1}i+q_{2}j+q_{3}k:i^{2}=j^{2}=k^{2}=ijk=-1,\;q_{0},q_{1},q_{2},q_{3}\in \mathbb{R}\right\}$. This defines an associative, non-commutative, division algebra.

In practice, quaternions are often referred to by their scalar and vectors parts as $q=\left(q_{0},\bar{q}\right)$, where *q*_0_∈ℝ and $\bar{q}={\left[q_{1},q_{2},q_{3}\right]}^{\text{T}}\in {\mathbb{R}}^{3}$. The properties of quaternion algebra are summarized in Table 1. Previous literature has defined quaternion multiplication as the multiplication between a 4 × 4 matrix and a vector in ℝ^4^, see for example Filipe and Tsiotras (2013b).

**Table 1:** Quaternion operations.

**Operation**	**Definition**
Addition	$a+b=\left(a_{0}+b_{0},ā+\bar{b}\right)$
Multiplication by a scalar	λ*a* = (λ*a*_0_, λ*a*)
Multiplication	$ab=\left(a_{0}b_{0}-ā\cdot \bar{b},a_{0}\bar{b}+b_{0}ā+ā\times \bar{b}\right)$
Conjugate	$a^{*}=\left(a_{0},-ā\right)$
Dot product	$a\cdot b=\left(a_{0}b_{0}+ā\cdot \bar{b},0_{3\times 1}\right)=\frac{1}{2}\left(a^{*}b+b^{*}a\right)$
Cross product	$a\times b=\left(0,a_{0}\bar{b}+b_{0}ā+ā\times \bar{b}\right)=\frac{1}{2}\left(ab-b^{*}a^{*}\right)$
Norm	$||a||=\sqrt{a\cdot a}$

Since any rotation can be described by three parameters, the unit norm constraint is imposed on quaternions for attitude representation. *Unit* quaternions are closed under multiplication, but not under addition. A quaternion describing the orientation of frame X with respect to frame Y, *q*_X/Y_, satisfies $q_{\text{X}/\text{Y}}^{*}q_{\text{X}/\text{Y}}=q_{\text{X}/\text{Y}}q_{\text{X}/\text{Y}}^{*}=1$, where 1 = (1, 0_3 × 1_). This quaternion can be constructed as $q_{\text{X}/\text{Y}}=\left(\cos\left(\phi /2\right),\bar{n}\sin\left(\theta /2\right)\right)$, where $\bar{n}$ and θ are the *unit* Euler axis, and Euler angle of the rotation, respectively. It is worth emphasizing that $q_{\text{Y}/\text{X}}^{*}=q_{\text{X}/\text{Y}}$, and that *q*_X/Y_ and −*q*_X/Y_ represent the same rotation. Furthermore, given quaternions *q*_Y/X_ and *q*_Z/Y_, the quaternion describing the rotation from *X* to *Z* is given by *q*_Z/X_ = *q*_Y/X_*q*_Z/Y_. This equation for composition of rotations corresponds to a Hamilton product convention as opposed to a Shuster convention, both of which are described in Sommer et al. (2018).

Three-dimensional vectors can also be interpreted as special cases of quaternions. Specifically, given ${\bar{s}}^{\text{X}}\in {\mathbb{R}}^{3}$, the coordinates of a vector expressed in frame X, its quaternion representation is given by $s^{\text{X}}=\left(0,{\bar{s}}^{\text{X}}\right)\in {ℍ}^{v}$, where ℍ^*v*^ is the set of *vector* quaternions defined as ${ℍ}^{v}≜\left\{\left(q_{0},\bar{q}\right)\in ℍ:\;q_{0}=0\right\}$ (see Filipe, 2014 for further information). The change of the reference frame on a vector quaternion is achieved by the adjoint operation, and is given by $s^{\text{Y}}=q_{\text{Y}/\text{X}}^{*}s^{\text{X}}q_{\text{Y}/\text{X}}$. Additionally, given *s*∈ℍ^*v*^, we can define the operation [·]^×^:ℍ^*v*^ → ℝ^4 × 4^ as

(1) $${\left[s\right]}^{\times }=\left[\begin{array}{cc} 0 & 0_{1\times 3}\\ 0_{3\times 1} & {\left[\bar{s}\right]}^{\times } \end{array}\right],\text{ where }{\left[\bar{s}\right]}^{\times }=\left[\begin{array}{ccc} 0 & -s_{3} & s_{2}\\ s_{3} & 0 & -s_{1}\\ -s_{2} & s_{1} & 0 \end{array}\right].$$

For quaternions $a=\left(a_{0},\bar{a}\right)$ and $b=\left(b_{0},\bar{b}\right)\in ℍ$, the left and right quaternion multiplication operators ${\left[\left[\cdot \right]\right]}_{\text{L}},{\left[\left[\cdot \right]\right]}_{\text{R}}:ℍ\to {\mathbb{R}}^{4\times 4}$ will be defined as

(2) $$ab≜{〚a〛}_{\text{L}}*b≜{〚b〛}_{\text{R}}*a,$$

where

(3) $${〚a〛}_{\text{L}}=\left[\begin{array}{cccc} a_{0} & -a_{1} & -a_{2} & -a_{3}\\ a_{1} & a_{0} & -a_{3} & a_{2}\\ a_{2} & a_{3} & a_{0} & -a_{1}\\ a_{3} & -a_{2} & a_{1} & a_{0} \end{array}\right]=\left[\begin{array}{cc} a_{0} & -{\bar{a}}^{\text{T}}\\ \bar{a} & a_{0}I_{3}+{\left[\bar{a}\right]}^{\times } \end{array}\right],$$

(4) $${〚b〛}_{\text{R}}=\left[\begin{array}{cccc} b_{0} & -b_{1} & -b_{2} & -b_{3}\\ b_{1} & b_{0} & b_{3} & -b_{2}\\ b_{2} & -b_{3} & b_{0} & b_{1}\\ b_{3} & b_{2} & -b_{1} & b_{0} \end{array}\right]=\left[\begin{array}{cc} b_{0} & -{\bar{b}}^{\text{T}}\\ \bar{b} & b_{0}I_{3}-{\left[\bar{b}\right]}^{\times } \end{array}\right].$$

The three-dimensional attitude kinematics evolve as

(5) $${\dot{q}}_{\text{X}/\text{Y}}=\frac{1}{2}q_{\text{X}/\text{Y}}\omega_{{}_{\text{X}/\text{Y}}}^{\text{X}}=\frac{1}{2}\omega_{{}_{\text{X}/\text{Y}}}^{\text{Y}}q_{{}_{\text{X}/\text{Y}}},$$

where $\omega_{\text{X}/\text{Y}}^{\text{Z}}≜\left(0,{\bar{\omega }}_{\text{X}/\text{Y}}^{\text{Z}}\right)\in {ℍ}^{v}$ and ${\bar{\omega }}_{\text{X}/\text{Y}}^{\text{Z}}\in {\mathbb{R}}^{3}$ is the angular velocity of frame X with respect to frame Y expressed in Z-frame coordinates.

### 2.2. Dual quaternions

The group of dual quaternion elements can be defined as

(6) $$\begin{array}{l} {\mathbb{Q}}_{d}≜\{-1,i,j,k,\epsilon ,\epsilon i,\epsilon j,\epsilon k:\;i^{2}=j^{2}=k^{2}=ijk=-1,\\ \;\epsilon i=i\epsilon ,\epsilon j=j\epsilon ,\epsilon k=k\epsilon ,\\ \;\epsilon \neq 0,\epsilon^{2}=0\}. \end{array}$$

Dual quaternion algebra arises as the algebra of the dual quaternion group ℚ_*d*_ over the field of real numbers, and is denoted as ℍ_*d*_. When dealing with the modeling of mechanical systems, it is convenient to define this algebra as ℍ_*d*_ = {***q*** = *q*_*r*_ + ϵ*q*_*d*_ : *q*_*r*_, *q*_*d*_ ∈ ℍ}, where ϵ is the dual unit. We call *q*_*r*_ the real part, and *q*_*d*_ the dual part of the dual quaternion **q**.

Filipe and Tsiotras (2014) and Filipe (2014) have laid out much of the groundwork in terms of the notation and the basic properties of dual quaternions. The main properties of dual quaternion algebra are listed in Table 2. Filipe and Tsiotras (2013b) also conveniently define a multiplication between matrices and dual quaternions that resembles the well-known matrix-vector multiplication by simply representing the dual quaternion coefficients as a vector in ℝ^8^.

**Table 2:** Dual quaternion operations.

**Operation**	**Definition**
Addition	***a*** + ***b*** = (*a*_*r*_ + *b*_*r*_) + ϵ(*a*_*d*_ + *b*_*d*_)
Multiplication by a scalar	λ***a*** = (λ*a*_*r*_) + ϵ(λ*a*_*d*_)
Multiplication	***ab*** = (*a*_*r*_*b*_*r*_) + ϵ(*a*_*d*_*b*_*r*_ + *a*_*r*_*b*_*d*_)
Conjugate	${\text{a}}^{*}=\left(a_{r}^{*}\right)+\epsilon \left(a_{d}^{*}\right)$
Dot product	$\text{a}\cdot \text{b}=\left(a_{r}\cdot b_{r}\right)+\epsilon \left(a_{d}\cdot b_{r}+a_{r}\cdot b_{d}\right)=\frac{1}{2}\left({\text{a}}^{*}\text{b}+{\text{b}}^{*}\text{a}\right)$
Cross product	$\text{a}\times \text{b}=\left(a_{r}\times b_{r}\right)+\epsilon \left(a_{d}\times b_{r}+a_{r}\times b_{d}\right)=\frac{1}{2}\left(\text{a}\text{b}-{\text{b}}^{*}{\text{a}}^{*}\right)$
Circle product	***a*** ° ***b*** = (*a*_*r*_·*b*_*r*_+*a*_*d*_·*b*_*d*_)+ϵ0
Swap	${\text{a}}^{s}=a_{d}+\epsilon a_{r}$
Norm	$||a||=\sqrt{a{}^{\circ}a}$
Vector part	$\text{vec}(\text{a})=\left(0,\bar{a_{r}}\right)+\epsilon \left(0,\bar{a_{d}}\right)$

Analogous to the set of vector quaternions ℍ^*v*^, we can define the set of vector dual quaternions as ${ℍ}_{d}^{v}≜\left\{\text{q}=q_{r}+\epsilon q_{d}:\;q_{r},q_{d}\in {ℍ}^{v}\right\}$. For vector dual quaternions we will define the skew-symmetric operator ${\left[\cdot \right]}^{\times }:{ℍ}_{d}^{v}\to {\mathbb{R}}^{8\times 8}$ as

(7) $${\left[s\right]}^{\times }≜\left[\begin{array}{cc} {\left[s_{r}\right]}^{\times } & 0_{4\times 4}\\ {\left[s_{d}\right]}^{\times } & {\left[s_{r}\right]}^{\times } \end{array}\right].$$

For dual quaternions ***a*** = *a*_*r*_ + ϵ*a*_*d*_ and ***b*** = *b*_*r*_ + ϵ*b*_*d*_ ∈ ℍ_*d*_, the left and right dual quaternion multiplication operators ${〚\cdot 〛}_{\text{L}},{〚\cdot 〛}_{\text{R}}:{ℍ}_{d}\to {\mathbb{R}}^{8\times 8}$ are defined as

(8) $$ab≜{〚a〛}_{\text{L}}⋆b≜{〚b〛}_{\text{R}}⋆a,$$

where

(9) $${〚a〛}_{\text{L}}=\left[\begin{array}{cc} {〚a_{r}〛}_{\text{L}} & 0_{4\times 4}\\ {〚a_{d}〛}_{\text{L}} & {〚a_{r}〛}_{\text{L}} \end{array}\right]\text{ and }{〚b〛}_{\text{R}}=\left[\begin{array}{cc} {〚b_{r}〛}_{\text{R}} & 0_{4\times 4}\\ {〚b_{d}〛}_{\text{R}} & {〚b_{r}〛}_{\text{R}} \end{array}\right].$$

The following lemma deals with the transformation invariance of the dual quaternion cross product operation.

**Lemma 1**. *The dual quaternion cross product is invariant to frame transformations. Specifically*,

(10) $$a^{\text{Y}}\times b^{\text{Y}}=\left(q_{\text{Y}/\text{X}}^{*}a^{\text{X}}q_{\text{Y}/\text{X}}\right)\times \left(q_{\text{Y}/\text{X}}^{*}b^{\text{X}}q_{\text{Y}/\text{X}}\right)=q_{\text{Y}/\text{X}}^{*}\left(a^{\text{X}}\times b^{\text{X}}\right)q_{\text{Y}/\text{X}}.$$

**Proof**. From the definition of the dual quaternion cross product given in Table *2*, we have that

(11) $$\begin{array}{l} q_{\text{Y}/\text{X}}^{*}\left(a^{\text{X}}\times b^{\text{X}}\right)q_{\text{Y}/\text{X}}=q_{\text{Y}/\text{X}}^{*}\left(a^{\text{X}}b^{\text{X}}-{\left(b^{\text{X}}\right)}^{*}{\left(a^{\text{X}}\right)}^{*}\right)q_{\text{Y}/\text{X}}\\ \;=q_{\text{Y}/\text{X}}^{*}a^{\text{X}}b^{\text{X}}q_{\text{Y}/\text{X}}-q_{\text{Y}/\text{X}}^{*}{\left(b^{\text{X}}\right)}^{*}{\left(a^{\text{X}}\right)}^{*}q_{\text{Y}/\text{X}}\\ \;=q_{\text{Y}/\text{X}}^{*}a^{\text{X}}q_{\text{Y}/\text{X}}q_{\text{Y}/\text{X}}^{*}b^{\text{X}}q_{\text{Y}/\text{X}}\\ \;-q_{\text{Y}/\text{X}}^{*}{\left(b^{\text{X}}\right)}^{*}q_{\text{Y}/\text{X}}q_{\text{Y}/\text{X}}^{*}{\left(a^{\text{X}}\right)}^{*}q_{\text{Y}/\text{X}}\\ \;=\left(q_{\text{Y}/\text{X}}^{*}a^{\text{X}}q_{\text{Y}/\text{X}}\right)\times \left(q_{\text{Y}/\text{X}}^{*}b^{\text{X}}q_{\text{Y}/\text{X}}\right)\\ \;=a^{\text{X}}\times b^{\text{X}}. \end{array}$$

The following lemma recasts the identity operation on a dual quaternion in terms of the left and right dual quaternion multiplication operations.

**Lemma 2**. *Given unit **q** ∈ ℍ_*d*_, the left and right dual quaternion multiplication matrix operators satisfy the following identities*:

(12) $$\begin{array}{l} {〚q〛}_{\text{L}}{〚q^{*}〛}_{\text{R}}{〚q^{*}〛}_{\text{L}}{〚q〛}_{\text{R}}=I_{8\times 8}\\ {〚q^{*}〛}_{\text{L}}{〚q〛}_{\text{R}}{〚q〛}_{\text{L}}{〚q^{*}〛}_{\text{R}}=I_{8\times 8} \end{array}$$

**Proof**. *To prove the first equality, let us apply the left-hand-side on the generic dual quaternion* **a** ∈ ℍ_*d*_ *and apply the definition of the multiplication matrix operators given in Equation (8) as*

(13) $$\begin{array}{l} {〚q〛}_{\text{L}}{〚q^{*}〛}_{\text{R}}{〚q^{*}〛}_{\text{L}}{〚q〛}_{\text{R}}⋆a={〚q〛}_{\text{L}}{〚q^{*}〛}_{\text{R}}{〚q^{*}〛}_{\text{L}}⋆aq\\ \;={〚q〛}_{\text{L}}{〚q^{*}〛}_{\text{R}}⋆\left(q^{*}aq\right)\\ \;={〚q〛}_{\text{L}}⋆\left(q^{*}aq\right)q^{*}\\ \;=q\left(q^{*}aq\right)q^{*}, \end{array}$$

and since qq^*^ = qq^*^ = **1**, the result follows. The second equality is proven analogously.

Since rigid body motion has six degrees of freedom, a dual quaternion needs two constraints to parameterize it. The dual quaternion describing the relative pose of frame B relative to I is given by $q_{\text{B}/\text{I}}=q_{\text{B}/\text{I},r}+\epsilon q_{\text{B}/\text{I},d}=q_{\text{B}/\text{I}}+\epsilon \frac{1}{2}q_{\text{B}/\text{I}}r_{\text{B}/\text{I}}^{\text{B}}$, where $r_{\text{B}/\text{I}}^{\text{B}}$ is the position quaternion describing the location of the origin of frame B relative to that of frame I, expressed in B-frame coordinates. It can be easily observed that *q*_B/I, *r*_·*q*_B/I, *r*_ = *1* and *q*_B/I, *r*_·*q*_B/I, *d*_ = 0, where $0=\left(0,\bar{0}\right)$, providing the two necessary constraints. Thus, a dual quaternion representing a pose transformation is a *unit* dual quaternion, since it satisfies ***q*** · ***q*** = ***q*** ^*^ **q** = **1**, where **1** ≜ *1* + ϵ0. Additionally, we also define **0** ≜ 0 + ϵ0.

Similar to the standard quaternion relationships, two dual quaternion transformations can be composed to yield a third one via dual quaternion multiplication as ***q***_*Z/X*_ = ***q***_*Y/X*_***q***_*Z/Y*_. Finally, the dual quaternion inverse is obtained using the conjugation operation, denoted as ${\text{q}}_{\text{Y}/\text{X}}^{*}={\text{q}}_{\text{X}/\text{Y}}$.

A useful equation is the generalization of the velocity of a rigid body in dual form, which contains both the linear and angular velocity components. The dual velocity of the Y-frame with respect to the Z-frame, expressed in X-frame coordinates, is defined as

(14) $$\omega_{\text{Y}/\text{Z}}^{\text{x}}=q_{\text{X}/\text{Y}}^{*}\omega_{\text{Y}/\text{Z}}^{\text{Y}}q_{\text{X}/\text{Y}}=\omega_{\text{Y}/\text{Z}}^{\text{x}}+\in \left(v_{\text{Y}/\text{Z}}^{\text{x}}+\omega_{\text{Y}/\text{Z}}^{\text{x}}\times r_{\text{Y}/\text{Z}}^{\text{x}}\right),$$

where $\omega_{\text{Y}/\text{Z}}^{\text{X}}=\left(0,{\bar{\omega }}_{\text{Y}/\text{Z}}^{\text{X}}\right)$ and $v_{\text{Y}/\text{Z}}^{\text{X}}=\left(0,{\bar{v}}_{\text{Y}/\text{Z}}^{\text{X}}\right)$, ${\bar{\omega }}_{\text{Y}/\text{Z}}^{\text{X}}$ and ${\bar{v}}_{\text{Y}/\text{Z}}^{\text{X}}\in {\mathbb{R}}^{3}$ are, respectively, the angular and linear velocity of the Y-frame with respect to the Z-frame expressed in X-frame coordinates, and $r_{\text{X}/\text{Y}}^{\text{X}}=\left(0,{\bar{r}}_{\text{X}/\text{Y}}^{\text{X}}\right)$, where ${\bar{r}}_{\text{X}/\text{Y}}^{\text{X}}\in {\mathbb{R}}^{3}$ is the position vector from the origin of the Y-frame to the origin of the X-frame expressed in X-frame coordinates. In particular, the dual velocity of a rigid body assigned to frame 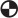
_i_ with respect to the inertial frame, expressed in 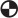
_i_-frame coordinates will be denoted 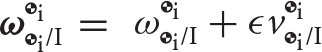.

In general, the dual quaternion kinematics can be expressed as Filipe and Tsiotras (2013b)

(15) $${\dot{q}}_{\text{X}/\text{Y}}=\frac{1}{2}q_{\text{X}/\text{Y}}\omega_{\text{X}/\text{Y}}^{\text{X}}=\frac{1}{2}\omega_{\text{X}/\text{Y}}^{\text{Y}}q_{\text{X}/\text{Y}}.$$

#### 2.2.1. Wrench and twists and their transformations using dual quaternions

**Figure 1:**
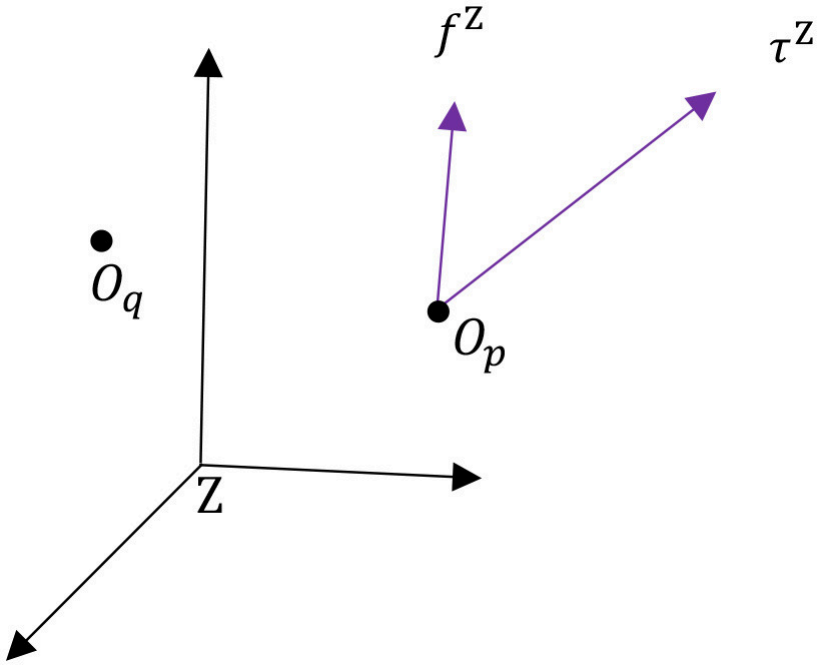
Wrench interpretation.

In order to take full advantage of the potential of dual quaternions in the context of dynamic modeling of multibody systems, we have to specify how forces and torques are shifted from one frame to another. This will allow us, for example, to easily shift the application of a reaction force at a joint onto the center of mass of a given body, among other applications. A wrench ${\text{W}}^{\text{Z}}\left(O_{p}\right)\in {ℍ}_{d}^{v}$ expressed in Z-frame coordinates can be expressed in terms of its components as

(16) $$W^{\text{Z}}\left(O_{p}\right)=f^{\text{Z}}+\epsilon \tau^{\text{Z}},$$

where $f^{\text{Z}}=\left(0,{\bar{f}}^{\text{Z}}\right),\;\tau^{\text{Z}}=\left(0,{\bar{\tau }}^{\text{Z}}\right)\in {ℍ}^{v}$ represent force and torque quaternions applied at point *O*_*p*_ as shown in Figure 1. Equivalently, we can describe the effect of *f*^Z^ and τ^Z^ about another point *O*_*q*_ as

(17) $$W^{\text{Z}}\left(O_{q}\right)=f^{\text{Z}}+\epsilon \left(\tau^{\text{Z}}+r_{\text{p}/\text{q}}^{\text{Z}}\times f^{\text{Z}}\right),$$

where the extra torque term is due to the moment arm from point *O*_*q*_ to point *O*_*p*_, captured by the position vector $r_{\text{p}/\text{q}}^{\text{Z}}$. Applying a frame transformation operation on a wrench about point *O*_X_ expressed in X-frame coordinates, given by ${\text{W}}^{\text{X}}\left(O_{\text{X}}\right)=f^{\text{X}}+\epsilon \tau^{\text{X}}$, yields the following expression

$$\begin{array}{l} W^{\text{Y}}\left(O_{\text{Y}}\right)=q_{\text{Y}/\text{X}}^{*}W^{\text{X}}\left(O_{\text{X}}\right)q_{\text{Y}/\text{X}}\\ \;={\left(q_{\text{Y}/\text{X}}+\epsilon \frac{1}{2}r_{\text{Y}/\text{X}}^{\text{X}}q_{\text{Y}/\text{X}}\right)}^{*}\left(f^{\text{X}}+\epsilon \tau^{\text{X}}\right)\left(q_{\text{Y}/\text{X}}+\epsilon \frac{1}{2}r_{\text{Y}/\text{X}}^{\text{X}}q_{\text{Y}/\text{X}}\right)\\ \;=\left(q_{\text{Y}/\text{X}}^{*}+\epsilon \frac{1}{2}q_{\text{Y}/\text{X}}^{*}r_{\text{Y}/\text{X}}^{\text{X}*}\right)\left(f^{\text{X}}+\epsilon \tau^{\text{X}}\right)\left(q_{\text{Y}/\text{X}}+\epsilon \frac{1}{2}r_{\text{Y}/\text{X}}^{\text{X}}q_{\text{Y}/\text{X}}\right)\\ \;=\left(q_{\text{Y}/\text{X}}^{*}-\epsilon \frac{1}{2}q_{\text{Y}/\text{X}}^{*}r_{\text{Y}/\text{X}}^{\text{X}}\right)\left(f^{\text{X}}+\epsilon \tau^{\text{X}}\right)\left(q_{\text{Y}/\text{X}}+\epsilon \frac{1}{2}r_{\text{Y}/\text{X}}^{\text{X}}q_{\text{Y}/\text{X}}\right)\\ \;=\left(q_{\text{Y}/\text{X}}^{*}-\epsilon \frac{1}{2}q_{\text{Y}/\text{X}}^{*}r_{\text{Y}/\text{X}}^{\text{X}}\right)\left(f^{\text{X}}q_{\text{Y}/\text{X}}+\epsilon \left(\tau^{\text{X}}q_{\text{Y}/\text{X}}+f^{\text{X}}\frac{1}{2}r_{\text{Y}/\text{X}}^{\text{X}}q_{\text{Y}/\text{X}}\right)\right)\\ \;=q_{\text{Y}/\text{X}}^{*}f^{\text{X}}q_{\text{Y}/\text{X}}-\epsilon \left(\frac{1}{2}q_{\text{Y}/\text{X}}^{*}r_{\text{Y}/\text{X}}^{\text{X}}f^{\text{X}}q_{\text{Y}/\text{X}}\right)\\ \;+\epsilon \left(q_{\text{Y}/\text{X}}^{*}\tau^{\text{X}}q_{\text{Y}/\text{X}}+q_{\text{Y}/\text{X}}^{*}f^{\text{X}}\frac{1}{2}r_{\text{Y}/\text{X}}^{\text{X}}q_{\text{Y}/\text{X}}\right)\\ \;=f^{\text{Y}}+\epsilon (\tau^{\text{Y}}+\frac{1}{2}q_{\text{Y}/\text{X}}^{*}f^{\text{X}}q_{\text{Y}/\text{X}}q_{\text{Y}/\text{X}}^{*}r_{\text{Y}/\text{X}}^{\text{X}}q_{\text{Y}/\text{X}}\\ \;-\frac{1}{2}q_{\text{Y}/\text{X}}^{*}r_{\text{Y}/\text{X}}^{\text{X}}q_{\text{Y}/\text{X}}q_{\text{Y}/\text{X}}^{*}f^{\text{X}}q_{\text{Y}/\text{X}})\\ \;=f^{\text{Y}}+\epsilon \left(\tau^{\text{Y}}+\frac{1}{2}f^{\text{Y}}r_{\text{Y}/\text{X}}^{\text{Y}}-\frac{1}{2}r_{\text{Y}/\text{X}}^{\text{Y}}f^{\text{Y}}\right)\\ \;=f^{\text{Y}}+\epsilon \left(\tau^{\text{Y}}+\frac{1}{2}f^{\text{Y}}r_{\text{Y}/\text{X}}^{\text{Y}}-\frac{1}{2}{\left(r_{\text{Y}/\text{X}}^{\text{Y}}\right)}^{*}{\left(f^{\text{Y}}\right)}^{*}\right), \end{array}$$

and by the definition of the cross product of two pure quaternion quantities given in Table 2, we get that

(18) $$\begin{array}{l} W^{\text{Y}}\left(O_{\text{Y}}\right)=q_{\text{Y}/\text{X}}^{*}W^{\text{X}}\left(O_{\text{X}}\right)q_{\text{Y}/\text{X}}\\ \;=f^{\text{Y}}+\epsilon \left(\tau^{\text{Y}}+f^{\text{Y}}\times r_{\text{Y}/\text{X}}^{\text{Y}}\right)\\ \;=f^{Y}+\epsilon \left(\tau^{\text{Y}}+r_{\text{X}/\text{Y}}^{\text{Y}}\times f^{\text{Y}}\right). \end{array}$$

The transformation described by Equation (18) implies that, given the dual force (e.g., force and torque) applied on a body at location *O*_X_, the equivalent wrench about a different location *O*_Y_ can be computed by using a simple frame transformation operation, commonly known as the *shifting law*. As expected, the transformation changes the reference frame in which the original (X-frame) force and torque are being expressed, but it also adds a torque term that arises due to the lever of the force *f*^X^ with respect to the new reference point *O*_Y_.

Equivalently, the following transformation of ${\text{W}}^{\text{Y}}\left(O_{\text{Y}}\right)=f^{\text{Y}}+\epsilon \tau^{\text{Y}}$ can be easily derived:

(19) $$\begin{array}{l} W^{\text{X}}\left(O_{\text{X}}\right)=q_{\text{Y}/\text{X}}W^{\text{Y}}\left(O_{\text{Y}}\right)q_{\text{Y}/\text{X}}^{*}\\ \;=f^{\text{X}}+\epsilon \left(\tau^{\text{X}}+r_{\text{Y}/\text{X}}^{\text{X}}\times f^{\text{X}}\right). \end{array}$$

Finally, when using wrenches, subscripts will be used to denote the source of, or a descriptor for, the wrench. For example, ${\text{W}}_{\text{ext}}^{\text{X}}\left(O_{p}\right)$ denotes that the source of the wrench is “ext,” which for our case denotes an external force and torque applied at the end effector of the robotic arm. It is worth emphasizing that the wrench transformation can be used to merely change the orientation of the frame on which the wrench is expressed, or to simply translate the origin, without re-orientating the axes.

The frame transformation relationships we have just derived not only apply to wrenches, but also to twists. Therefore, given the twist ${\text{s}}^{\text{X}}=s_{r}^{\text{X}}+\epsilon s_{d}^{\text{X}}$ the adjoint transformation can be described by

(20) $$\begin{array}{l} s^{\text{Y}}=q_{\text{Y}/\text{X}}^{*}s^{\text{X}}q_{\text{Y}/\text{X}}\\ \;=s_{r}^{\text{Y}}+\epsilon \left(s_{d}^{\text{Y}}+s_{r}^{\text{Y}}\times r_{\text{Y}/\text{X}}^{\text{Y}}\right)\\ \;=s_{r}^{\text{Y}}+\epsilon \left(s_{d}^{\text{Y}}+r_{\text{X}/\text{Y}}^{\text{Y}}\times s_{r}^{\text{Y}}\right). \end{array}$$

Equivalently, given ${\text{s}}^{\text{Y}}=s_{r}^{\text{Y}}+\epsilon s_{d}^{\text{Y}}$, the inverse adjoint transformation is described by

(21) $$\begin{array}{l} s^{\text{X}}=q_{\text{Y}/\text{X}}s^{\text{Y}}q_{\text{Y}/\text{X}}^{*}\\ \;=s_{r}^{\text{X}}+\epsilon \left(s_{d}^{\text{X}}+s_{r}^{\text{X}}\times r_{\text{X}/\text{Y}}^{\text{X}}\right)\\ \;=s_{r}^{\text{X}}+\epsilon \left(s_{d}^{\text{X}}+r_{\text{Y}/\text{X}}^{\text{X}}\times s_{r}^{\text{X}}\right). \end{array}$$

#### 2.2.2. Dual inertia matrix, dual momentum and 6-DOF rigid body dynamics

The dual inertia matrix for a rigid body computed about its center of mass can be as follows Filipe and Tsiotras (2013b) 
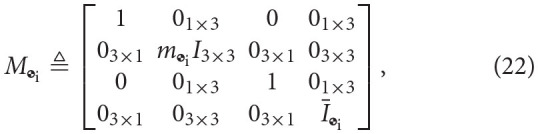


where *m*_
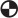
_i__ ∈ ℝ is the mass of the *i*-th body, 
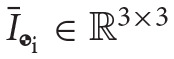
 is the rigid body mass inertia matrix of the *i*-th body, and *I*_3 × 3_ is the 3-by-3 identity matrix.

The dual momentum of body *i* computed about its center of mass and expressed in local frame 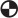
_i_ can be defined as 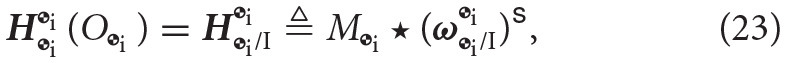


where the ⋆ operator can be interpreted as conventional matrix-vector multiplication when the dual quaternion is represented as a vector in ℝ^8^. The kinetic energy for a rigid body can be computed as 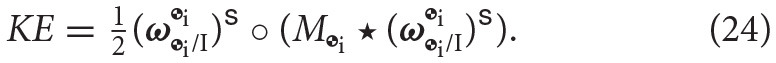


We can also define the matrix operator H(·):ℝ^8 × 8^ → ℝ^8 × 8^ to eliminate the swap operation in a matrix-dual quaternion multiplication. Applied on a matrix multiplying a dual quaternion **a** ∈ ℍ_*d*_, we have that

(25) $$\text{H}\left(M\right)⋆a≜M⋆a^{\text{s}}.$$

In block form, this operator acts on *M* ∈ ℝ^8 × 8^, composed of blocks $M_{1},M_{2}\in {\mathbb{R}}^{8\times 4}$, as follows

(26) $$\text{H}(M)=\text{H}\left(\left[M_{1},M_{2}\right]\right)=\left[M_{2},M_{1}\right],$$

and, in particular, it acts on the dual inertia matrix *M*_
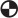
_i__ as 
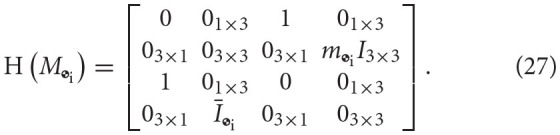


Therefore, we can also write the dual momentum as 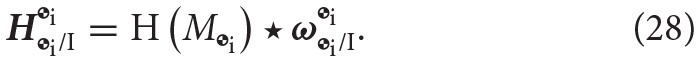


The following lemma deals with the invertibility of H(*M*_
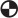
_i__).

**Lemma 3**. *The inverse of* H(*M*_
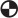
_i__), H(*M*_
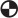
_i__)^−1^, *exists and is given by* 
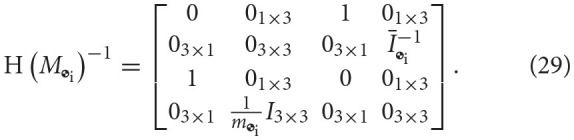


**Proof**. Through evaluation, 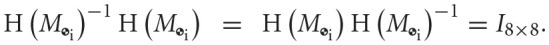.

For a multibody system *S*, with B rigid bodies whose centers of mass are located at 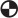
_i_, Equation (23) can be generalized to 
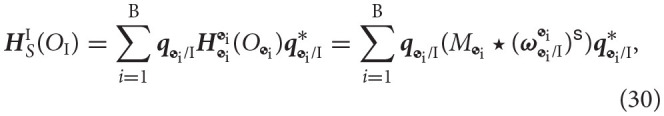


yielding the dual momentum of the system computed about the origin of the inertial frame and expressed in I-frame coordinates. The kinetic energy of Equation (24) can be generalized as 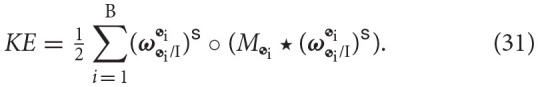


From Equation (23), we can compute the 6-DOF dynamic equations of motion of body *i* as 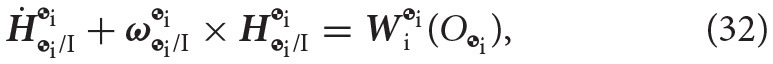


or equivalently, 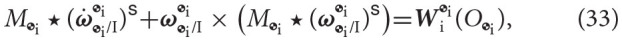


where 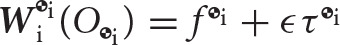 is the net wrench applied on body *i* about its center of mass.

## 3. Multibody system modeling using dual quaternions

This section aims to provide a generalized dual quaternion framework to model the kinematics and the dynamics of a multibody system that contains joints of the following types:

1. Revolute (R)
2. Prismatic (P)
3. Spherical (S)
4. Cylindrical (C)
5. Cartesian (U).

The approach is aimed toward characterizing spacecraft with one or more serial robotic arms having varying lengths. The framework, in fact, will hold for robotic arms that branch out themselves, while preserving a rooted tree structure, with the satellite base being the root.

As in previous sections, we will use roman variables for frames, subscripts and superscripts of physical quantities. We will use standard math font for the labeling of physical components, like bodies and joints. For example, body *i* will have its center of mass at 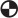
_i_.

### 3.1. Variable definition and conventions

We will model the spacecraft as a graph G(v,e), where *v* is the number of vertices, and *e* represents the number of edges. This graph, in particular, will correspond to that of a directed and rooted tree with arborescent branching, that is, a graph with tree structure where direction of the edges matters, and these in general point away from the root.

**Figure 2:**
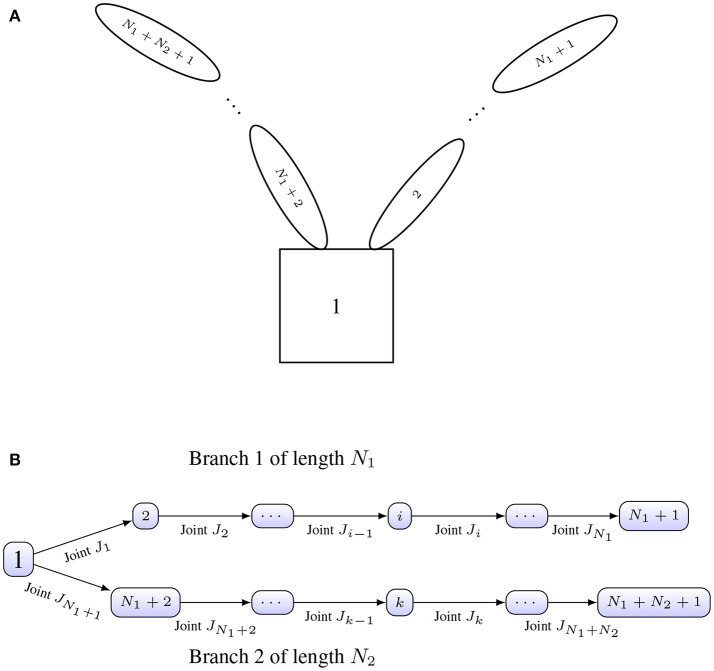
**(A)** Conceptual spacecraft architecture. **(B)** Architecture as rooted tree with labeled joints.

For our specific application, the nodes of the graph will be the different rigid bodies composing the serial manipulator(s), and the edges will be the different joints of the manipulator(s). Figure 2A shows an example of the labeling for the different rigid bodies composing a two-arm configuration on a satellite. The same configuration is shown in Figure 2B with the labeling of the vertices (nodes) and edges accordingly.

As is common in graph theory, matrices will aid in the description of the system's topology. Two matrices will be particularly useful in this generalization: the incidence matrix, denoted by *C*, and the branch termination vector, denoted as *T*. The incidence matrix contains information about the connectivity between the joints and the bodies. The columns of the incidence matrix represent rigid bodies, while the rows represent joints. Thus, entry *C*_*ij*_ indicates the relationship between joint *i* and rigid body *j* as follows

(34) $${\left(C\right)}_{i,j}=c_{ij}≜\{\begin{array}{ll} 1, & \text{ if joint }i\text{ is proximal, body is distal, }\\ 0, & \text{ if joint }i\text{ is not connected to body }j,\\ -1, & \text{ if joint }i\text{ is distal, body }j\text{ is proximal,} \end{array}$$

where the relative positions are with respect to the satellite base.

The branch termination vector, *T* denotes whether the given body is the end of a branch. The body will most likely be an end-effector and external wrenches due to interaction with the environment may be applied on it. We define the vector *T* as

(35) $${\left(T\right)}_{i}=t_{i}≜\{\begin{array}{ll} 1, & \text{ if body }i\text{ ends a branch, }\\ 0, & \text{ otherwise}. \end{array}$$

We will define the functions *N*(·), *P*(·) as follows. Given a row or column of matrix *C*, or vector *T*, they output the indices of the “−1” entries, and the indices of the “+1” entries, respectively. Additionally, we will use the notation *C*(:, *j*) to identify the *j*-th column of *C*, *C*(*i*, :) to identify the *i*-th row of matrix *C*. It is worth emphasizing that each row will contain exactly one “−1” entry and exactly one “+1” entry, although, in general, columns can have several “−1” or “1” entries^1^.

**Figure 3:**
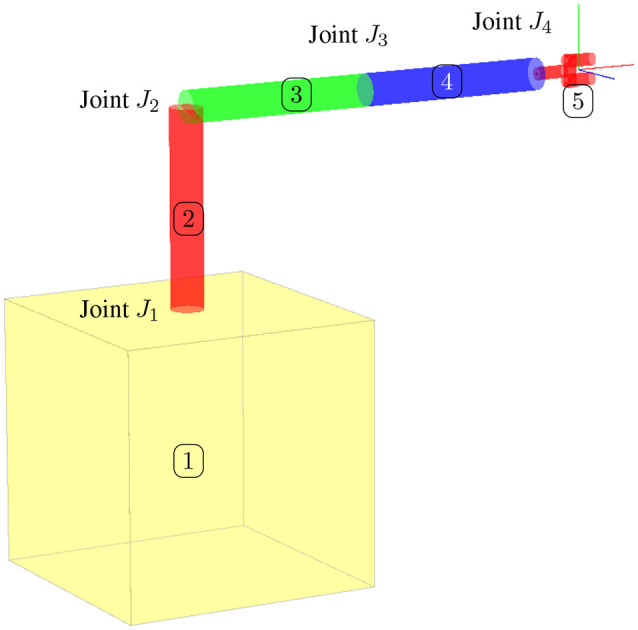
Robotic arm configuration on a satellite base.

**Example 1**. The incidence and branch termination matrices for the architecture shown in Figure 3 are given by 
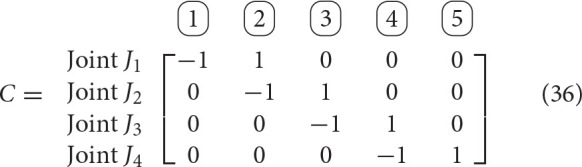
 
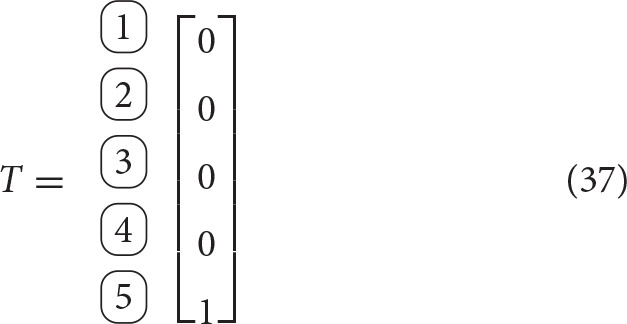


As example of the usage of the functions *N*(·) and *P*(·), we have

(38) N(C(1,:))=N(row 1 of matrix C)={1},

(39) P(C(1,:))=P(row 1 of matrix C)={2},

(40) P(T)=P(vector T)={5}.

Let *N*_*i*_ be the length of branch *i*, *d*_*i*_ be the degrees of freedom of joint *J*_*i*_, J the total number of joints, and B the total number of rigid bodies. Therefore, B = 1+J, and $\text{J}=\sum_{i\;\in \;\text{Branches}}N_{i}$. Using this notation, matrix *C* ∈ ℝ^J × B^ and vector *T* ∈ ℝ^B^. We will define D as the total number of degrees of freedom added by the joints, which can be computed as $\text{D}=\sum_{i\;\in \;\text{Joints}}d_{i}$. Exploiting the duality between degrees of freedom at a joint, *d*_*i*_, and the dimensionality of the reaction wrench, *r*_*i*_, we will define $\text{R}=\sum_{i\;\in \;\text{Joints}}r_{i}=\sum_{i\;\in \;\text{Joints}}6-d_{i}$.

The vector **y** ∈ ℝ^8B^ is defined as the collection of dual velocities, given by 
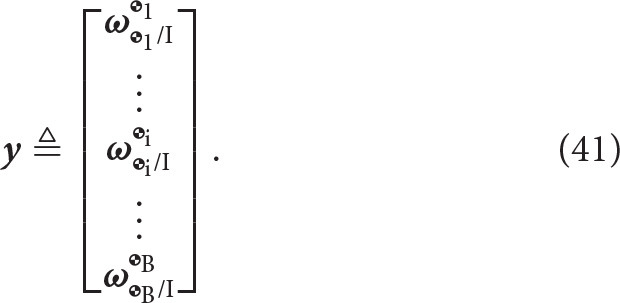


The vector of generalized coordinates Γ ∈ ℝ^D^ represents the generalized coordinates of the joints and it is defined as

(42) $$\Gamma ≜\left[\begin{array}{l} \Gamma_{{\text{J}}_{1}}\\ \vdots \\ \Gamma_{{\text{J}}_{\text{i}}}\\ \vdots \\ \Gamma_{{\text{J}}_{\text{J}}} \end{array}\right],$$

where the form of Γ_J_i__ is dependent upon the type of joint *J*_*i*_. Table 3 lists the parametrization used for each type of joint. Here it is worth noting that the generalized coordinates parametrize the motion of the *i* frame (fixed to the distal body with respect to the joint) with respect to the proximal body, which is captured by the index k, where k = *N*(*C*(*i*, :)). In particular, S joints are modeled with an Eulerian 3-2-1 (yaw ψ, pitch θ, roll ϕ) rotation sequence for uniformity with other types of joints, even though these can be better parametrized by quaternions to avoid issues with singularities during the evaluation of the kinematics.

**Table 3:** Generalized coordinates Γ_J_i__ for joint *J*_*i*_ depending on its type.

**Joint type**	**Generalized coordinate parametrization**	***d*_*i*_ (DOF)**
R	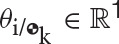	1
P	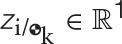	1
S	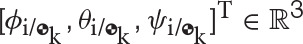	3
C	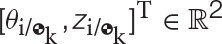	2
U	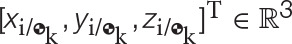	3
	k = *N*(*C*(*i*, :))	

Thus, the state vector for any given spacecraft-robotic arm configuration will be given by 
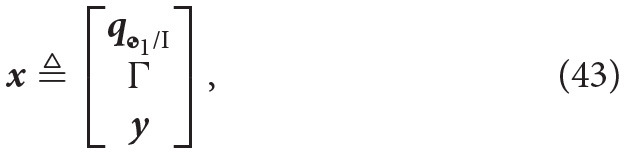


where **q**_
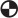
_1_/*I*_ ∈ ℍ_*d*_ is the pose of the base.

**Figure 4:**
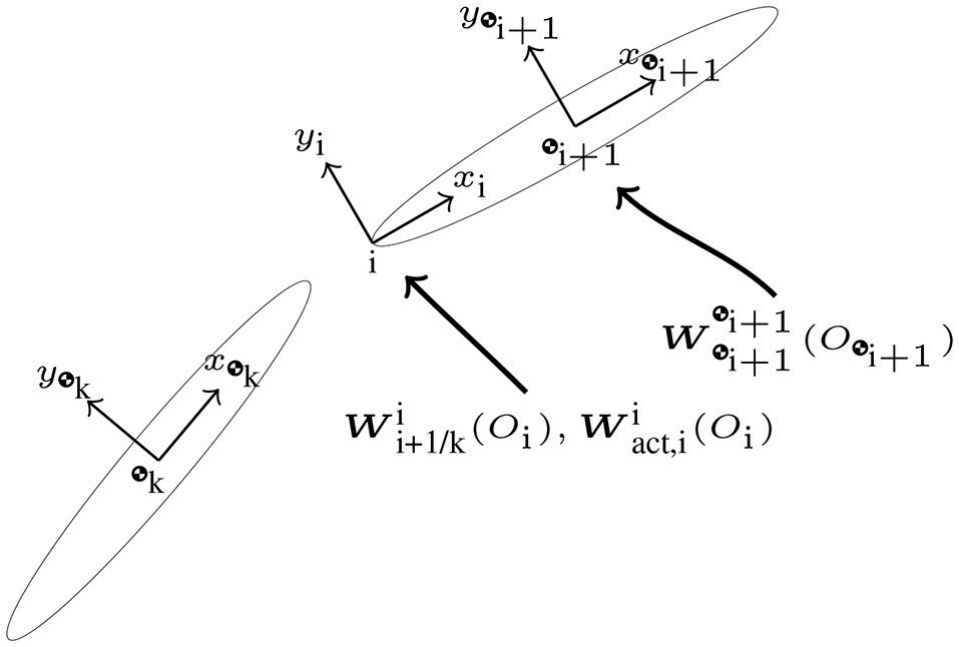
Body frame labeling and wrench definition at joint *J*_*i*_ between bodies *i*+1 and *k* = *N*(*C*(*i*, :)).

Figure 4 shows joint *J*_*i*_ with its associated frame i; the frame 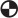
_i+1_, which has the same orientation as frame i but its origin is at the center of mass of body *i* + 1; and the frame at the center of mass of the proximal body denoted by 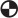
_k_, where k = *N*(*C*(*i*, :)). The origin of the i frame is positioned at the physical interface between the two adjoining bodies. Figure 4 also shows three types of wrenches. The reaction and actuation wrenches appear at the joint, with their point of application being the origin of the joint frame *O*_i_, and their coordinates expressed in the i frame. We additionally show the body wrench 
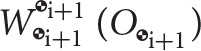
. Joint actuation wrenches ${\text{W}}_{\text{act},\text{i}}^{\text{i}}\left(O_{\text{i}}\right)$ induce motion about the degrees of freedom of the joint. Reaction wrenches ${\text{W}}_{\text{i}+1/\text{k}}^{\text{i}}\left(O_{\text{i}}\right)$ arise due to physical constraints at the joints, and they are dual in nature to the joint actuation wrenches. Body wrenches, which are assumed to act at the center of mass of the body, come from control sources or other natural phenomena such as gravitational effects, or atmospheric drag, all appropriately transformed to the center of mass through the shifting law. For an example on the use of the naming convention for frames and wrenches, the reader is referred to the Appendix.

It will be assumed that the degrees of freedom of the joints are along the *Z*_i_-axis, which is a common assumption in the field of robotics, while the *X*_i_ and *Y*_i_ axes can be selected according to any predetermined set of rules, such as those laid out in Chapter 5 of Jazar (2010). The exceptions are the Cartesian and spherical joints, both of which have three degrees of freedom, and for which an orientation of the axes must be assumed a priori. For the cartesian joint, the local coordinate system is defined such that it is parallel to the physical axes of motion. For the spherical joint, one suggestion is to define the *X*_i_ pointing toward the *i*+1th rigid body, while the *Y*_i_ and *Z*_i_ complete the orthogonal axis system.

We will define $T\in {\mathbb{R}}^{\text{R}}$, the collection of reduced reaction wrenches, as

(44) $$T≜\left[\begin{array}{l} {\tilde{W}}_{2/1}^{1}\left(O_{1}\right)\\ \vdots \\ {\tilde{W}}_{\text{i}+1/\text{N}(\text{C}(\text{i},:))}^{\text{i}}\left(O_{\text{i}}\right)\\ {\tilde{W}}_{\text{i}+2/\text{N}(\text{C}(\text{i}+1,:))}^{\text{i}+1}\left({\text{O}}_{\text{i}+1}\right)\\ \vdots \\ {\tilde{W}}_{\text{B}+1/\text{N}(\text{C}(\text{B},:))}^{\text{B}}\left({\text{O}}_{\text{B}}\right) \end{array}\right],$$

where ${\tilde{\text{W}}}_{\text{i}+1/\text{N}\left(\text{C}\left(\text{i},:\right)\right)}^{\text{i}}\in {\mathbb{R}}^{r_{i}}$ is obtained from ${\text{W}}_{\text{i}+1/\text{N}\left(\text{C}\left(\text{i},:\right)\right)}^{\text{i}}\in {ℍ}_{d}$ by eliminating the entries that correspond to the generalized coordinate of the joint, since there are no reaction forces or torques applied on the bodies about that generalized coordinate. In general, we can obtain ${\tilde{\text{W}}}_{\text{i}+1/\text{N}\left(\text{C}\left(\text{i},:\right)\right)}^{\text{i}}$ from ${\text{W}}_{\text{i}+1/\text{N}\left(\text{C}\left(\text{i},:\right)\right)}^{\text{i}}$ using the relationship ${\text{W}}_{\text{i}+1/\text{N}\left(\text{C}\left(\text{i},:\right)\right)}^{\text{i}}=V_{i}{\tilde{\text{W}}}_{\text{i}+1/\text{N}\left(\text{C}\left(\text{i},:\right)\right)}^{\text{i}}$, the form of the matrix $V_{i}\in {\mathbb{R}}^{8\times r_{i}}$ depending on the type of joint. Table 4 lists the general wrench ${\text{W}}_{\text{i}+1/\text{N}\left(\text{C}\left(\text{i},:\right)\right)}^{\text{i}}$, the reduced wrench ${\tilde{\text{W}}}_{\text{i}+1/\text{N}\left(\text{C}\left(\text{i},:\right)\right)}^{\text{i}}$, and the mapping matrix *V*_*i*_ for each of the joints considered. The matrix *E*_π(1, 2, 3, 4, 5, 6, 7, 8;*i*)_ is formed by removing rows π(1, 2, 3, 4, 5, 6, 7, 8;*i*) from the eight-by-eight. The function π(·;*i*) selects an ordered subset of {1, 2, 3, 4, 5, 6, 7, 8} based on the type of joint *i*. The matrices Λ_*i*_ are provided for compactness, as they will be used in a future section as a way of eliminating a degree of freedom from a constraint equation for a given type of joint. Also, for completion purposes, we provide the form of the actuation wrenches in Table 5 and its corresponding mapping matrix from reduced actuation wrenches, identified by *V*_act, i_.

**Table 4:** Form of reduced reaction wrenches for different joint types.

**Joint type**	**${\text{W}}_{\text{i+1}/\text{i}}^{\text{i}}$**	**${\tilde{\text{W}}}_{\text{i+1}/\text{i}}^{\text{i}}$**	***V*_i_**	**Λ_*i*_**
R	$\left(0,{\left[f_{x},f_{y},f_{z}\right]}^{\text{T}}\right)+\epsilon \left(0,{\left[\tau_{x},\tau_{y},0\right]}^{\text{T}}\right)$	${\left[f_{x},f_{y},f_{z},\tau_{x},\tau_{y}\right]}^{\text{T}}$	$E_{158}^{\text{T}}$	*E*_145_
P	$\left(0,{\left[f_{x},f_{y},0\right]}^{\text{T}}\right)+\epsilon \left(0,{\left[\tau_{x},\tau_{y},\tau_{z}\right]}^{\text{T}}\right)$	${\left[f_{x},f_{y},\tau_{x},\tau_{y},\tau_{z}\right]}^{\text{T}}$	$E_{145}^{\text{T}}$	*E*_158_
S	$\left(0,{\left[f_{x},f_{y},f_{z}\right]}^{\text{T}}\right)+\epsilon \left(0,{\left[0,0,0\right]}^{\text{T}}\right)$	${\left[f_{x},f_{y},f_{z}\right]}^{\text{T}}$	$E_{15678}^{\text{T}}$	*E*_12345_
C	$\left(0,{\left[f_{x},f_{y},0\right]}^{\text{T}}\right)+\epsilon \left(0,{\left[\tau_{x},\tau_{y},0\right]}^{\text{T}}\right)$	${\left[f_{x},f_{y},\tau_{x},\tau_{y}\right]}^{\text{T}}$	$E_{1458}^{\text{T}}$	*E*_1458_
U	$\left(0,{\left[0,0,0\right]}^{\text{T}}\right)+\epsilon \left(0,{\left[\tau_{x},\tau_{y},\tau_{z}\right]}^{\text{T}}\right)$	${\left[\tau_{x},\tau_{y},\tau_{z}\right]}^{\text{T}}$	$E_{12345}^{\text{T}}$	*E*_15678_

**Table 5:** Form of actuation wrenches for different joint types.

**Joint type**	**${\text{W}}_{\text{act},\text{i}}^{\text{i}}$**	***V*_act, i_**
R	$\left(0,{\left[0,0,0\right]}^{\text{T}}\right)+\epsilon \left(0,{\left[0,0,\tau_{z}\right]}^{\text{T}}\right)$	$E_{1234567}^{\text{T}}$
P	$\left(0,{\left[0,0,f_{z}\right]}^{\text{T}}\right)+\epsilon \left(0,{\left[0,0,0\right]}^{\text{T}}\right)$	$E_{1235678}^{\text{T}}$
S	$\left(0,{\left[0,0,0\right]}^{\text{T}}\right)+\epsilon \left(0,{\left[\tau_{x},\tau_{y},\tau_{z}\right]}^{\text{T}}\right)$	$E_{12345}^{\text{T}}$
C	$\left(0,{\left[0,0,f_{z}\right]}^{\text{T}}\right)+\epsilon \left(0,{\left[0,0,\tau_{z}\right]}^{\text{T}}\right)$	$E_{123567}^{\text{T}}$
U	$\left(0,{\left[f_{x},f_{y},f_{z}\right]}^{\text{T}}\right)+\epsilon \left(0,{\left[0,0,0\right]}^{\text{T}}\right)$	$E_{15678}^{\text{T}}$

### 3.2. Kinematics

The kinematics of the system are fully characterized by the kinematics of the satellite base, and the kinematics of the joint generalized coordinates. The pose of the satellite base evolves as 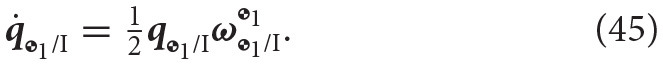


The joint dual velocity expressed in joint coordinates can be determined from 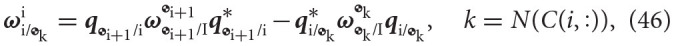


while the generalized coordinates of the joints can be determined to evolve as 
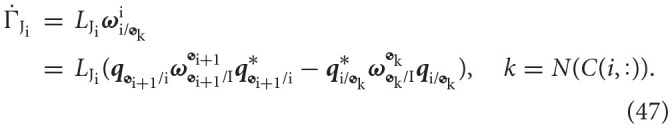


The matrix *L*_J_i__ depends on the joint type, and these are listed in Table 6.

**Table 6:** Mapping matrix from angular velocity to generalized coordinates.

**Joint type**	***L*_J_i__**
R	[0, 0, 0, 1, 0, 0, 0, 0]
P	[0, 0, 0, 0, 0, 0, 0, 1]
S	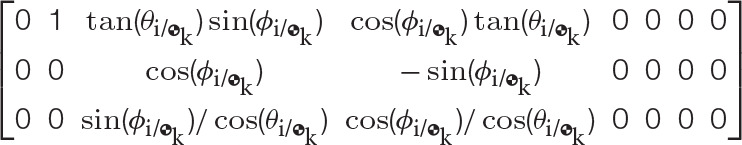
C	[0, 0, 0, 1, 0, 0, 0, 0 0, 0, 0, 0, 0, 0, 0, 1]
U	[0, 0, 0, 0, 0, 1, 0, 0 0, 0, 0, 0, 0, 0, 1, 0 0, 0, 0, 0, 0, 0, 0, 1]

Furthermore, from Equation (46), we can derive an acceleration-level relationship at each joint given by 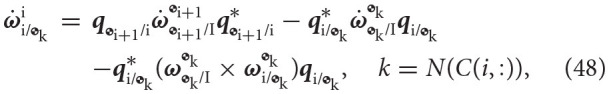


resulting in 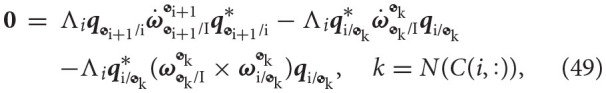


where we have used the fact that 
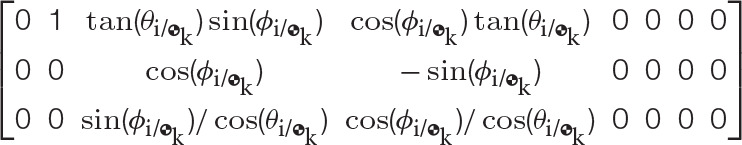
, by construction of Λ_*i*_, defined in Table 4.

### 3.3. Dynamics

We will now generalize the rigid body Newton-Euler. We will show that the equations of motion can be cast in the form

(50) $$\left[\begin{array}{c} S_{11}S_{12}\\ S_{21}S_{22} \end{array}\right]\left[\begin{array}{c} \dot{y}\\ T \end{array}\right]=\left[\begin{array}{c} {ℬ}_{1}\\ {ℬ}_{2} \end{array}\right].$$

We will define each of the blocks $S_{11}\in {\mathbb{R}}^{8\text{B}\times 8\text{B}}$, $S_{12}\in {\mathbb{R}}^{8\text{B}\times \text{R}}$, $S_{21}\in {\mathbb{R}}^{\text{R}\times 8\text{B}}$, $S_{22}\in {\mathbb{R}}^{\text{R}\times \text{R}}$, $B_{1}\in {\mathbb{R}}^{8\text{B}}$, and $B_{2}\in {\mathbb{R}}^{\text{R}}$ independently.

The block $S_{11}$ is composed of the dual inertia matrix for each of the bodies. It is given by

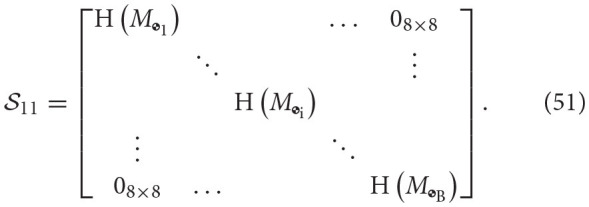



Notice that since this matrix is block diagonal, its inverse can be easily computed as the inverse of its sub-blocks, which exist as proven in Lemma 3. Thus, in cases when there are no moving mechanical components, fluid slosh, or fuel consumption, the inverse of the inverse of its sub-blocks, which exist as proven in Lemma 3. Thus, in cases when there are no moving mechanical components, fluid slosh, or fuel consumption, the inverse of $S_{11}$ can be pre-computed and stored in memory to speed up computations. The block $S_{22}\in {\mathbb{R}}^{\text{R}\times \text{R}}$ represents the effect of the reaction wrenches on the constraint equations. Since wrenches do not appear in the constraint equations, this block is composed of zeros. Explicitly, this block is given by

(52) $$S_{22}=0_{\text{R}\times \text{R}}.$$

The block $S_{12}\in {\mathbb{R}}^{8\text{B}\times \text{R}}$ couples the reaction wrenches with the dynamics of each body. These wrenches initially appear on the right-hand side of the Newton-Euler. The point of application of the wrench and the frame of reference are shifted to the center of mass of the body for which the equation is being derived. The matrix is composed of blocks of size ${\left(S_{12}\right)}_{ij}\in {\mathbb{R}}^{8\times r_{j}}$, corresponding to the attachment of body *i* to joint *j*, where each of these blocks is specified as 
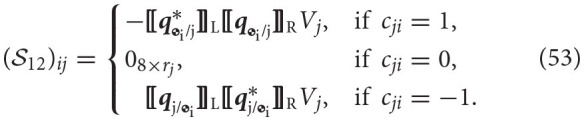


The form of matrix *V*_*j*_ depends on the type of joint as was detailed in Table 4. The block $S_{21}\in {\mathbb{R}}^{\text{R}\times 8\text{B}}$ introduces the dual accelerations of each body into the constraint equations. The matrix is composed of blocks ${\left(S_{21}\right)}_{ij}\in {\mathbb{R}}^{r_{i}\times 8}$, corresponding to the constraint at joint *i* and its relationship with body *j* as described by Equation (49). These sub-blocks are specified as 
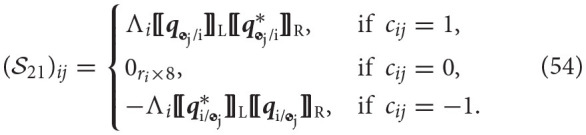


The form of matrix Λ_*i*_ depends on the type of joint and it is provided in Table 4.

The vector $B_{1}\in {\mathbb{R}}^{8\text{B}}$ corresponds to the right hand side of the Newton-Euler. In particular, it contains the non-linear term **ω**×(*M*⋆**ω**^*s*^), the known wrenches applied at the center of mass, and the wrenches due to joint actuation. If the body ends a branch, it is assumed that it can interact with the environment at a specific point in the body. This is included in $B_{1}$ as well through “external” wrenches. External wrenches for branch *i* will be assumed to act at frame G_i_, the frame assigned to the end-effector of branch-terminating body *i*, and they will be denoted by $W_{ext,i}^{G_{i}}(O_{G_{i}})$. The vector is composed of sub-vectors ${\left(B_{1}\right)}_{i}\in {\mathbb{R}}^{8}$ given by 
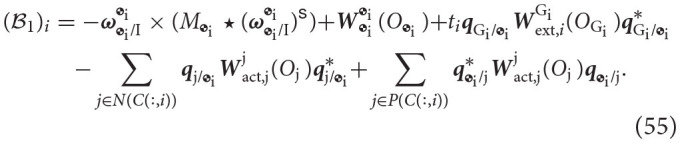


The vector $B_{2}\in {\mathbb{R}}^{\text{R}}$ corresponds to the right-hand-side of the constraint equations for each of the joints. In particular, it contains a cross term of dual velocities that arises when taking the derivative of the dual velocity constraint to yield a dual acceleration constraint, detailed in Equation (49). The vector is composed of sub-vectors ${\left(B_{2}\right)}_{i}\in {\mathbb{R}}^{r_{i}}$, given by 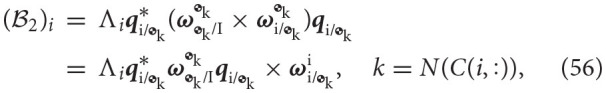


where in the last equality we used the invariance of the dual quaternion cross product, proven in Lemma 1.

Finally, since $S_{11}$ is always invertible and $S_{22}=0_{\text{R}\times \text{R}}$, we can avoid inverting the large matrix on the left-hand-side of Equation (50) by using the Schur complement. Thus, if

(57) $$S≜\left[\begin{array}{c} S_{11}S_{12}\\ S_{21}S_{22} \end{array}\right]=\left[\begin{array}{cc} S_{11} & S_{12}\\ S_{21} & 0_{\text{R}\times \text{R}} \end{array}\right]$$

we define the Schur complement of block $S_{11}$ as $S/S_{11}≜-S_{21}S_{11}^{-1}S_{12}$. Therefore, the inverse of S is given by

(58) $$S^{-1}=\left[\begin{array}{cc} S_{11}^{-1}+S_{11}^{-1}S_{12}{\left(S/S_{11}\right)}^{-1}S_{21}S_{11}^{-1} & -S_{11}^{-1}S_{12}{\left(S/S_{11}\right)}^{-1}\\ -{\left(S/S_{11}\right)}^{-1}S_{21}S_{11}^{-1} & {\left(S/S_{11}\right)}^{-1} \end{array}\right].$$

Hence, we can solve for the unknowns as

(59) $$\left[\begin{array}{l} \dot{y}\\ T \end{array}\right]=S^{-1}\left[\begin{array}{l} {ℬ}_{1}\\ {ℬ}_{2} \end{array}\right],$$

which upon expansion, yields

(60) $$\begin{array}{l} T={\left(S_{21}S_{11}^{-1}S_{12}\right)}^{-1}\left(S_{21}S_{11}^{-1}{ℬ}_{1}-{ℬ}_{2}\right),\\ \;\dot{y}=-S_{11}^{-1}S_{12}T+S_{11}^{-1}{ℬ}_{1}\\ \;=-S_{11}^{-1}S_{12}{\left(S_{21}S_{11}^{-1}S_{12}\right)}^{-1}\left(S_{21}S_{11}^{-1}{ℬ}_{1}-{ℬ}_{2}\right)+S_{11}^{-1}{ℬ}_{1}. \end{array}$$

### 3.4. Locking or prescribing joint motion

In some instances, it is desirable to lock a certain degree a freedom or prescribe its generalized coordinate, while still being able to determine the reaction wrenches produced by this motion. Additionally, knowledge of the required actuation wrench can provide insight into the holding torque that a given motor must provide, or exert during specific smaneuvers. A straight-forward modification of the equations provided herein can yield this information.

Let the admissible dual velocity and acceleration of the prescribed-motion for joint *J*_*i*_ be given by 
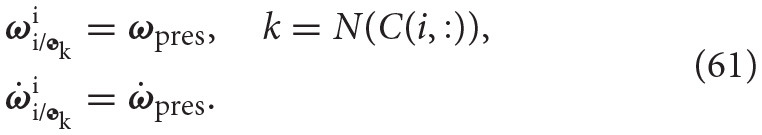


The generalized speed is still mapped as follows 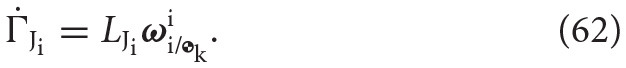


Assuming knowledge of the proximal body's dual acceleration 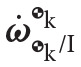, which must be solved for in tandem with all other dual accelerations and reaction wrenches, and since all velocity-level quantities are known, the distal body's dual velocity and acceleration are fully described by the kinematic relationships 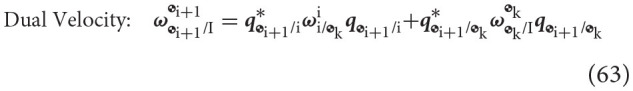 
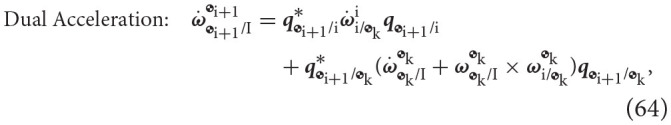


both of which can be easily derived from Equations (46) and (48). Since the dual acceleration 
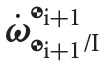
 is no longer an unknown, we must remove the corresponding equations from the system of equations presented in Equation (50). To do this, we remove 
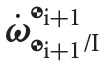
 from the vector of unknowns **ẏ**, and block-matrices ${\left(S_{11}\right)}_{\left\{:,i+1\right\}}$, ${\left(S_{21}\right)}_{\left\{:,i+1\right\}}$, which are the corresponding coefficients of 
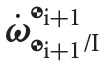
 that appear in both Newton-Euler. For the sake of exposition, let us rename these modified variables as $\hat{ẏ}$, ${\hat{S}}_{11}$, and ${\hat{S}}_{21}$.

Next, we need to manipulate the modified Newton-Euler. In general terms, this equation is given by 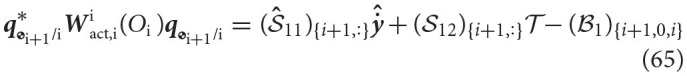


and 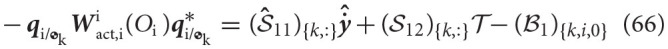


where we have defined 
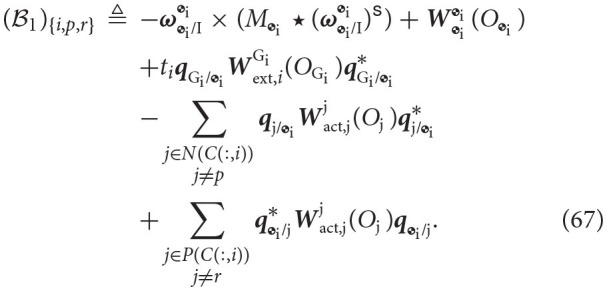


By manipulating Equations (65) and (66), we obtain 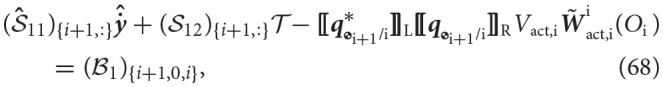


and 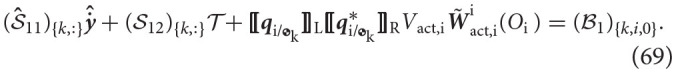


Further manipulation of Equation (68) allows clearing ${\tilde{\text{W}}}_{\text{act},\text{i}}^{\text{i}}\left(O_{\text{i}}\right)$ of transformations as 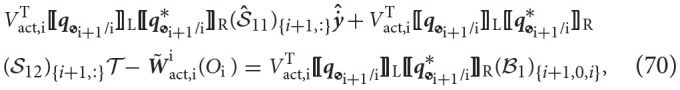


where we have used Lemma 2 and the fact that $V_{\text{act},\text{i}}^{\text{T}}V_{\text{act},\text{i}}=I_{d_{i}\times d_{i}}$ for ${\tilde{\text{W}}}_{\text{act},\text{i}}^{\text{i}}\left(O_{\text{i}}\right)\in {\mathbb{R}}^{d_{i}}$.

The resulting system of equations will be of the form

(71) $$\left[\begin{array}{ccc} ϒ{\hat{S}}_{11} & ϒS_{12} & S_{\text{act},\text{i},1}\\ {\hat{S}}_{21} & S_{22} & S_{\text{act},\text{i},2} \end{array}\right]\left[\begin{array}{c} \hat{\dot{y}}\\ T\\ {\tilde{W}}_{\text{act},\text{i}}^{\text{i}}\left(O_{\text{i}}\right) \end{array}\right]=\left[\begin{array}{c} ϒ{\hat{ℬ}}_{1}\\ {ℬ}_{2} \end{array}\right].$$

Here we have that

(72) $$S_{\text{act},\text{i},\text{2}}=0_{\text{R}\times d_{i}},$$

while $S_{\text{act},\text{i},1}\in {\mathbb{R}}^{\left(8\left(\text{B}-1\right)+d_{i}\right)\times d_{i}}$ is described by 
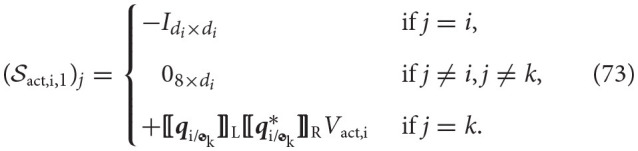


The vectors ${\hat{B}}_{1}$ and $B_{1}$ are identical, except the (*i*+1)-th and *k*-th entries, which are computed as

(74) $$\begin{array}{l} {\left({\hat{ℬ}}_{1}\right)}_{i+1}:={\left({ℬ}_{1}\right)}_{\left\{i+1,0,i\right\}}\\ \;{\left({\hat{ℬ}}_{1}\right)}_{k}:={\left({ℬ}_{1}\right)}_{\{k,i,0\}.} \end{array}$$

Additionally, the block diagonal matrix Υ is described as 
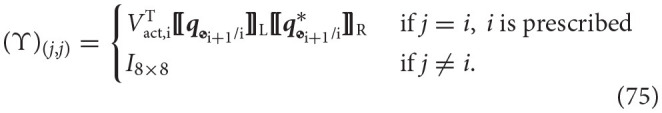


It is worth emphasizing that the resulting matrix

(76) $$\left[\begin{array}{ccc} ϒ{\hat{S}}_{11} & ϒS_{12} & S_{\text{act},\text{i},1}\\ {\hat{S}}_{21} & S_{22} & S_{\text{act},\text{i},2} \end{array}\right]$$

belongs to ${\mathbb{R}}^{\left(8\left(\text{B}-1\right)+R+d_{i}\right)\times \left(8\left(\text{B}-1\right)+R+d_{i}\right)}$ and thus, it is square and invertible.

### 3.5. Framework summary

**Algorithm** **1** provides a detailed description of how to implement the kinematics and dynamics framework introduced in the previous sections.

**Algorithm 1:**
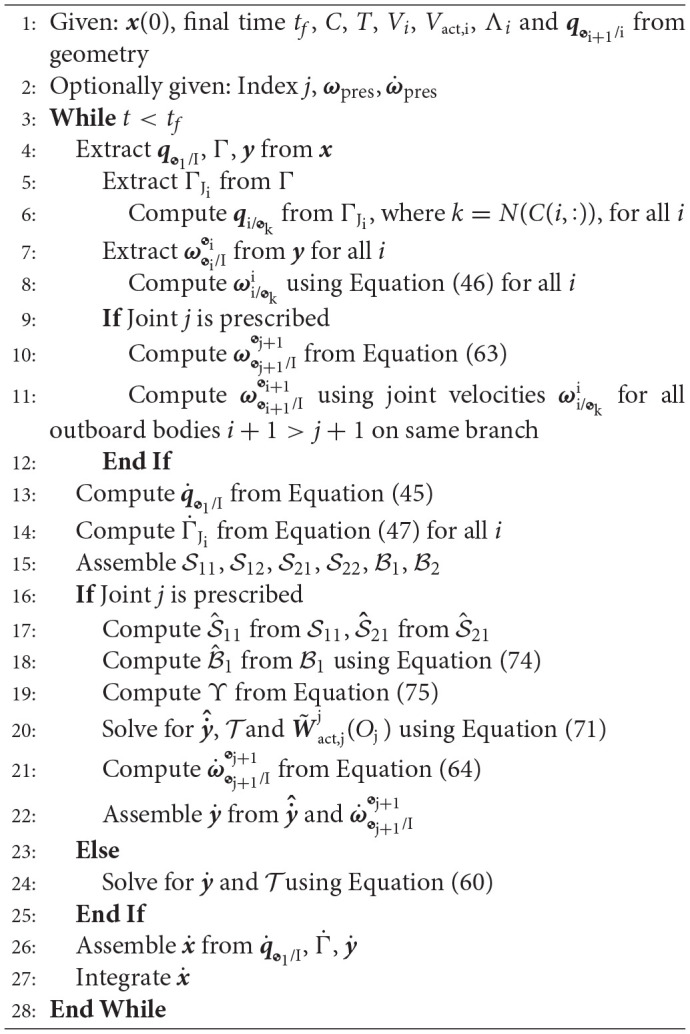
**Kinematics and dynamics of spacecraft-mounted robotic systems**.

## 4. Evaluation of numerical performance

We studied the performance of the algorithm on the satellite shown in Figure 3 without the end-effector. The inertias for the four different bodies were chosen as *M*_
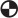
1_ = diag (1, 10, 10, 10, 1, 50, 50, 50) [kg, kg · m^2^], *M*_
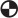
2_ = diag (1, 5, 5, 5, 1, 2, 2, 1) [kg, kg · m^2^], *M*_
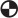
3_ = diag (1, 5, 5, 5, 1, 1, 2, 2) [kg, kg · m^2^], and *M*_
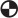
4_ = diag (1, 5, 5, 5, 1, 1, 2, 2) [kg, kg · m^2^]. The geometry of the system was chosen as 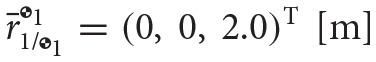, 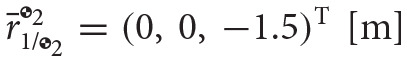, 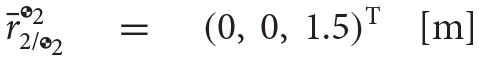, 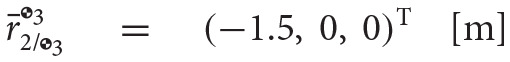, 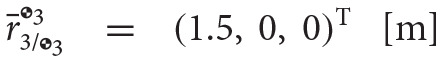, 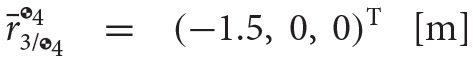, and 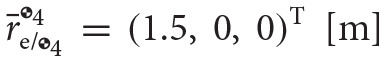, where the orientation of the frames for each of the bodies can be found in Chapter 5 of Valverde (2018).

The simulation was run using MATLAB R2017a's ODE45. The integrator's option AbsTol (absolute tolerance) was set to 1 × 10^−14^ and RelTol (relative tolerance) was set to 2.220 × 10^−14^; the final time was set to *t*_*f*_ = 70 *s*. To evaluate center of mass, linear momentum, and angular momentum conservation, only internal (joint) wrenches were applied. The generalized forces on the wrenches

(77) $$\begin{array}{l} W_{\text{act},1}^{1}\left(O_{0}\right)=0+\epsilon \left(0,{\left[0,0,{\left({\bar{\tau }}_{\text{act},1}\right)}_{z}\right]}^{\text{T}}\right)\\ W_{\text{act},2}^{2}\left(O_{1}\right)=0+\epsilon \left(0,{\left[0,0,{\left({\bar{\tau }}_{\text{act},2}\right)}_{z}\right]}^{\text{T}}\right)\\ W_{\text{act},3}^{3}\left(O_{2}\right)=0+\epsilon \left(0,{\left[0,0,{\left({\bar{\tau }}_{\text{act},3}\right)}_{z}\right]}^{\text{T}}\right), \end{array}$$

were set to

(78) $$\begin{array}{l} {\left({\bar{\tau }}_{\text{act},1}\right)}_{z}=\{\begin{array}{ll} 0.5\text{sin}(t-2)\text{N}, & 2s<t<5s\\ 0, & \text{ otherwise } \end{array}\\ {\left({\bar{\tau }}_{\text{act}2}\right)}_{z}=\{\begin{array}{ll} 0.5\text{sin}(t-10)\text{N}, & 10s<t<12s\\ 0, & \text{ otherwise } \end{array}\\ {\left({\bar{\tau }}_{\text{act},3}\right)}_{z}=\{\begin{array}{ll} 0.5\text{sin}(t-20)\text{N}, & 20s<t<22s\\ 0, & \text{ otherwise}. \end{array} \end{array}$$

**Figure 5:**
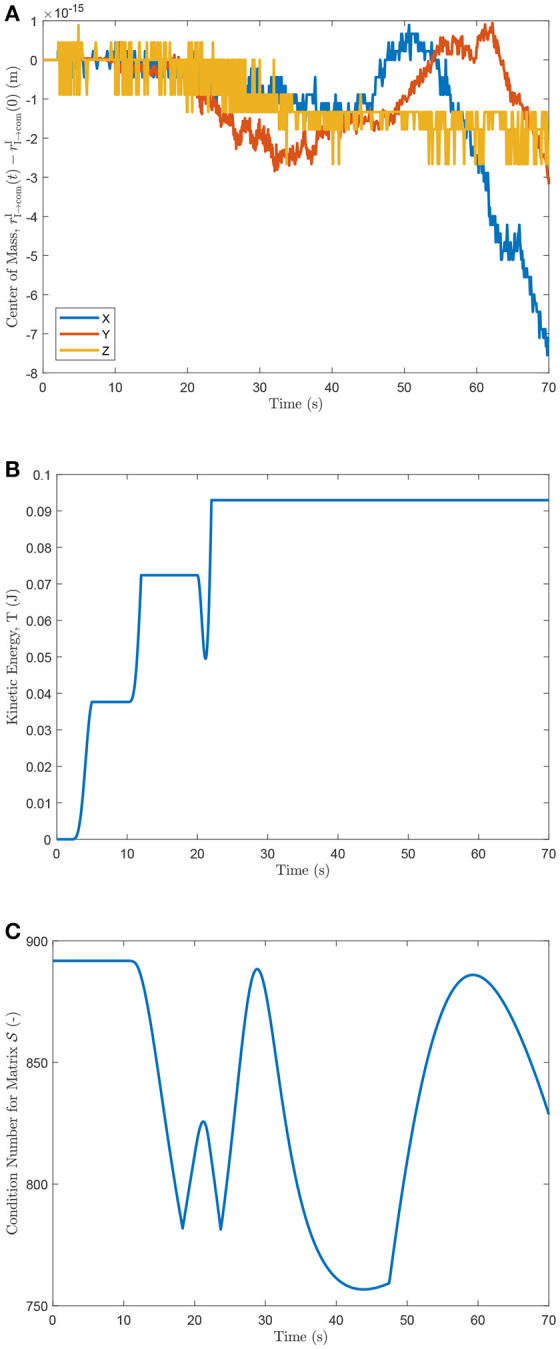
**(A)** Center of mass deviation from initial position, **(B)** kinetic energy of the system, and **(C)** condition number of S.

The deviation of the center of mass of the system with respect to its initial position is shown in Figure 5A. The total kinetic energy of the system is shown in Figure 5B, and the condition number for matrix S, described in Equation (57), is plotted in Figure 5C at every time step.

**Figure 6:**
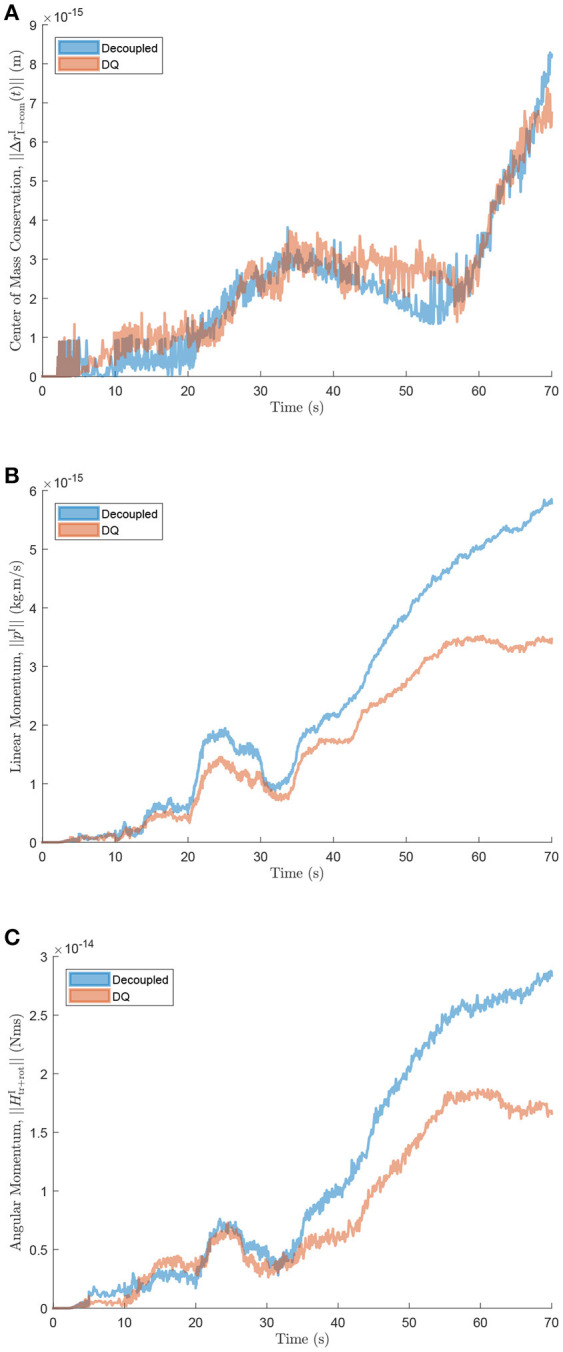
**(A)** Change in center of mass location, **(B)** linear, and **(C)** angular momentum comparison between decoupled and dual quaternion formulations.

The equations of motion were derived for the same architecture using classical Newton-Euler techniques as in the framework proposed by Stoneking Stoneking (2007), where the rotational dynamics are decoupled from the translational dynamics. The implementation required deriving the constraint equations for revolute joints, since these are not explicitly addressed by Stoneking. The numerical performance differences between the dual quaternion approach (DQ), and the decoupled formulation (Decoupled) of the dynamics, were evaluated for the same set of inputs. Figure 6A shows the comparison of the norm of the change of the center of mass of the system with respect to its initial position as a function of time. Next, the conservation of the linear and angular momenta of both systems is compared as shown in Figures 6B,C. As expected, the dual quaternion formulation possesses a numerical advantage since it more naturally accounts for the coupling between the rigid bodies' translational and rotational motion.

## 5. Conclusion

In this paper we have provided an intuitive approach to derive the dynamics of a satellite with a rooted-tree configuration with different joint types, including revolute, prismatic, spherical, cylindrical, and cartesian joints using dual quaternions. The approach exploits the structure of the Newton-Euler form of the dynamical equations of motion for a rigid body in dual quaternion form, allowing for the determination of the reaction wrenches at the joints. The different nature of the joints is captured by simple changes in the mapping matrices associated with each joint, and not through a fundamental change in the form of the equations - an advantage provided by the coupled nature of the kinematic and dynamic relationships expressed in terms of dual quaternions. The proposed framework can be particularly beneficial during proximity operations of a robotic servicing mission. Combining existing dual quaternion-based pose-tracking controllers with the proposed dual quaternion framework for the modeling of the multibody robotic servicer allows for the use of a unified algebra to model the different phases, ranging from navigation, to grappling and servicing.

## Author contributions

The content of this article was developed as part of AV's Ph.D. thesis, in close collaboration with the PT.

### Conflict of interest statement

The authors declare that the research was conducted in the absence of any commercial or financial relationships that could be construed as a potential conflict of interest.

## Figures and Tables

**Figure 7:**
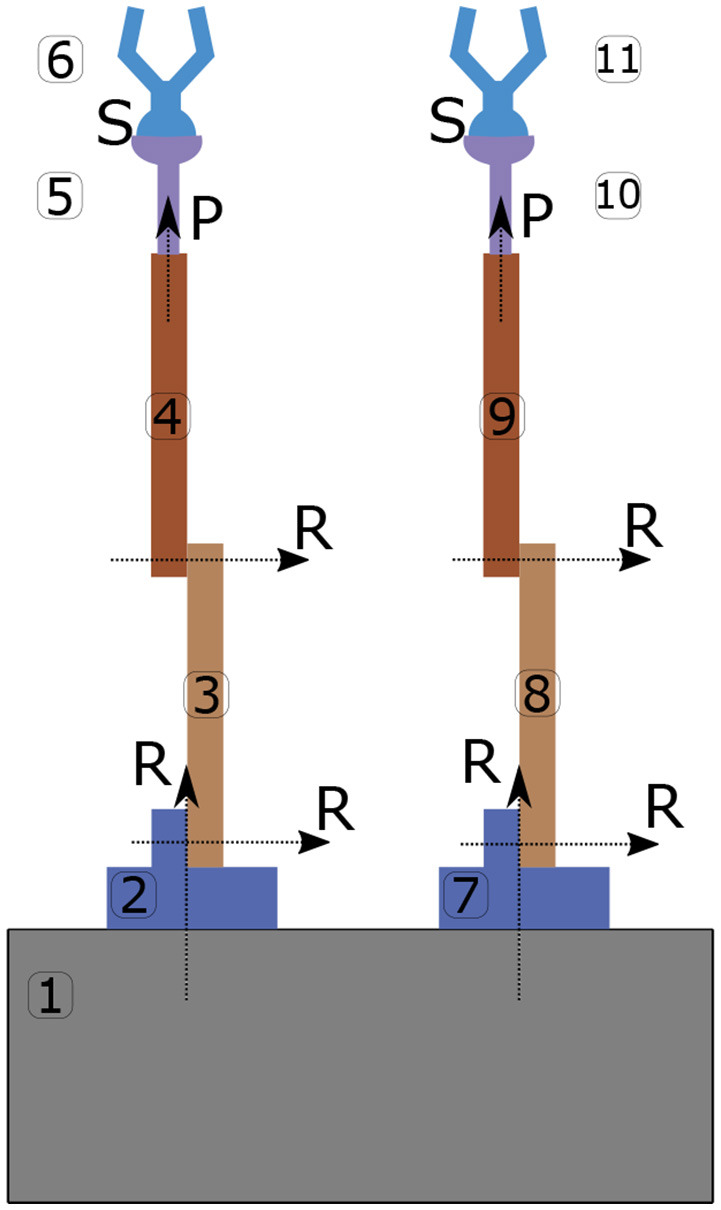
Proposed architecture with joint types in nominal configuration and body labeling.

**Figure 8:**
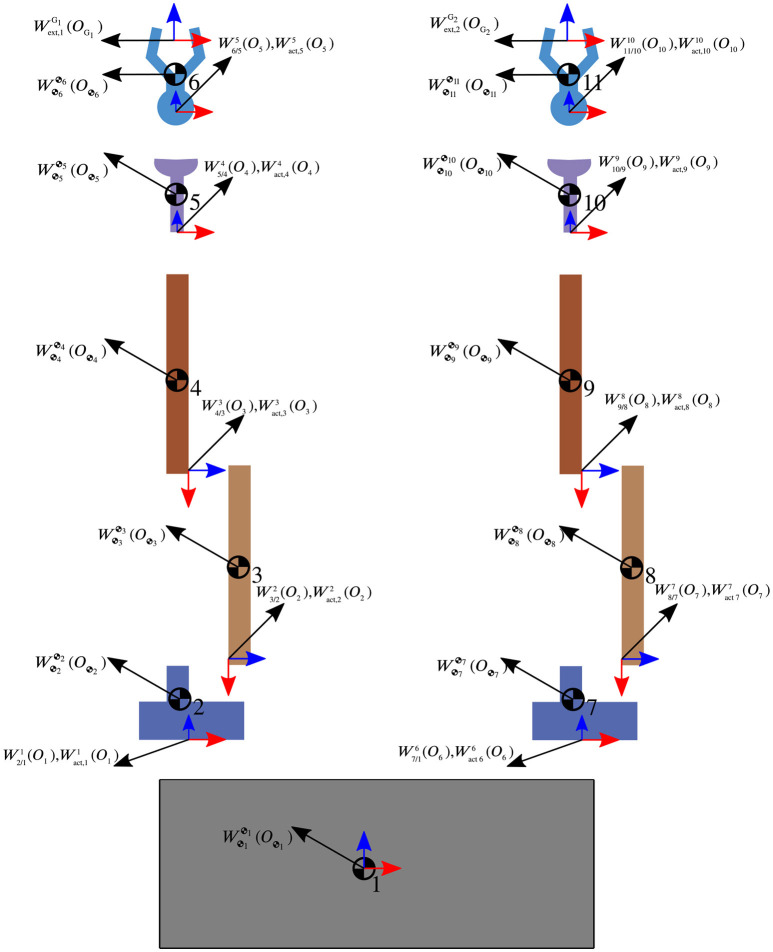
Coordinate system definition and wrenches for two arm satellite architecture.

## Footnotes

^1^The column corresponding to the satellite base will only have “−1” values, since no joint is proximal. The columns corresponding to end-effector bodies will only possess “+1” values since end-effectors are all distal with respect to their corresponding joint.

## Appendix:

### Labeling example on two arm manipulator

We present an example of a spacecraft with two robotic arms, each with five links and three different types of joints. The example aims to further familiarize the reader with the proposed notation. The architecture of the satellite is shown in Figure 7. Figure 8 shows a schematic of the coordinate frames and the wrenches defined during implementation of the proposed framework. Reaction wrenches and actuation wrenches are assumed positive as applied on the body on which they are shown, and negative on the proximal body relative to the joint.

## References

[B1] Bishop B. Gargano R. Sears A. Karpenko M. (2015). Rapid maneuvering of multi-body dynamic systems with optimal motion compensation. Acta Astronaut. 117, 209–221. 10.1016/j.actaastro.2015.07.035

[B2] Brodsky V. Shoham M. (1999). Dual numbers representation of rigid body dynamics. Mech. Mach. Theory 34, 693–718. 10.1016/S0094-114X(98)00049-4

[B3] Carignan C. R. Akin D. L. (2000). The reaction stabilization of on-orbit robots. IEEE Control Syst. Mag. 20, 19–33. 10.1109/37.887446

[B4] Dooley J. R. McCarthy J. M. (1993). On the geometric analysis of optimum trajectories for cooperating robots using dual quaternion coordinates, in Proceedings 1993 IEEE International Conference on Robotics and Automation, Vol. 1 (Atlanta, GA), 1031–1036.

[B5] Dubanchet V. Saussié D. Alazard D. Bérard C. Peuvédic C. L. (2015). Modeling and control of a space robot for active debris removal. CEAS Space J. 7, 203–218. 10.1007/s12567-015-0082-4

[B6] Dubowsky S. Vance E. E. Torres M. A. (1989). The control of space manipulators subject to spacecraft attitude control saturation limits, in Proceedings of the NASA Conference on Space Telerobotics, Vol. 4 (Pasadena, CA), 409–418.

[B7] Featherstone R. (2008). Rigid Body Dynamics Algorithms. Boston, MA: Springer-Verlag.

[B8] Featherstone R. Orin D. (2000). Robot dynamics: equations and algorithms, in Proceedings 2000 IEEE International Conference on Robotics and Automation, Vol. 1 (San Francisco, CA), 826–834.

[B9] Filipe N. (2014). Nonlinear Pose Control and Estimation for Space Proximity Operations: An Approach Based on Dual Quaternions. PhD thesis, Georgia Institute of Technology.

[B10] Filipe N. Kontitsis M. Tsiotras P. (2015). Extended Kalman filter for spacecraft pose estimation using dual quaternions. J. Guidance Control Dyn. 38, 1625–1641. 10.2514/1.G000977

[B11] Filipe N. Tsiotras P. (2013a). Rigid body motion tracking without linear and angular velocity feedback using dual quaternions, in European Control Conference (Zürich), 329–334.

[B12] Filipe N. Tsiotras P. (2013b). Simultaneous position and attitude control without linear and angular velocity feedback using dual quaternions, in Proceedings of the 2013 American Control Conference (Washington, DC), 4815–4820.

[B13] Filipe N. Tsiotras P. (2014). Adaptive position and attitude-tracking controller for satellite proximity operations using dual quaternions. J. Guidance Control Dyn. 38, 566–577. 10.2514/1.G000054

[B14] Hooker W. W. (1970). A set of r dynamical attitude equations for an arbitrary n-Body satellite having r rotational degrees of freedom. AIAA J. 8, 1205–1207. 10.2514/3.5873

[B15] Jain A. (1991). Unified formulation of dynamics for serial rigid multibody systems. J. Guidance Control Dyn. 14, 531–542. 10.2514/3.20672

[B16] Jazar R. N. (2010). Theory of Applied Robotics: Kinematics, Dynamics, and Control. New York, NY: Springer Science & Business Media.

[B17] Lee J. Grey M. X. Ha S. Kunz T. Jain S. Ye Y. (2018). DART: dynamic animation and robotics toolkit. J. Open Source Softw. 3:500 10.21105/joss.00500

[B18] Longman R. W. Lindbergt R. E. Zedd M. F. (1987). Satellite-mounted robot manipulators - new kinematics and reaction moment compensation. Int. J. Robot. Res. 6, 87–103. 10.1177/027836498700600306

[B19] Mohan A. Saha S. K. (2007). A recursive, numerically stable, and efficient simulation algorithm for serial robots. Multibody Syst. Dyn. 17, 291–319. 10.1007/s11044-007-9044-8

[B20] Moosavian S. A. A. Papadopoulos E. (2004). Explicit dynamics of space free-flyers with multiple manipulators via spacemaple. Adv. Robot. 18, 223–244. 10.1163/156855304322758033

[B21] Papadopoulos E. Dubowsky S. (1990). On the nature of control algorithms for space manipulators, in Proceedings 1990 IEEE International Conference on Robotics and Automation, Vol. 2 (Cincinnati, OH), 1101–1108.

[B22] Papadopoulos E. Dubowsky S. (1991a). Coordinated manipulator/spacecraft motion control for space robotic systems, in Proceedings 1991 IEEE International Conference on Robotics and Automation (Sacramento, CA), 1696–1701.

[B23] Papadopoulos E. Dubowsky S. (1991b). On the nature of control algorithms for free-floating space manipulators. IEEE Trans. Robot. Autom. 7, 750–758. 10.1109/70.105384

[B24] Rodriguez G. Jain A. Kreutz-Delgado K. (1991). A spatial operator algebra for manipulator modeling and control. Int. J. Robot. Res. 10, 371–381. 10.1177/027836499101000406

[B25] Rodriguez G. Jain A. Kreutz-Delgado K. (1992). Spatial operator algebra for multibody system dynamics. J. Astronaut. Sci. 40, 27–50.

[B26] Saha S. K. (1999). Dynamics of serial multibody systems using the decoupled natural orthogonal complement matrices. J. Appl. Mech. 66, 986–996. 10.1115/1.2791809

[B27] Saha S. K. Shah S. V. Nandihal P. V. (2013). Evolution of the DeNOC-based dynamic modelling for multibody systems. Mech. Sci. 4, 1–20. 10.5194/ms-4-1-2013

[B28] Seo D. (2015). Fast adaptive pose tracking control for satellites via dual quaternion upon non-certainty equivalence principle. Acta Astronaut. 115, 32–39. 10.1016/j.actaastro.2015.05.013

[B29] Sherman M. Rosenthal D. (2001). SD/FAST. Available online at: https://support.ptc.com/support/sdfast/index.html

[B30] Sommer H. Gilitschenski I. Bloesch M. Weiss S. Siegwart R. Nieto J. (2018). Why and how to avoid the flipped quaternion multiplication. Aerospace 5, 1–15. 10.3390/aerospace5030072

[B31] Stoneking E. (2007). Newton-Euler dynamic equations of motion for a multi-body spacecraft, in AIAA Guidance, Navigation and Control Conference and Exhibit (Hilton Head, SC).

[B32] Stoneking E. (2013). Implementation of Kane's method for a spacecraft composed of multiple rigid bodies, in AIAA Guidance, Navigation, and Control (GNC) Conference (Boston, MA).

[B33] Umetani Y. Yoshida K. (1989). Resolved motion rate control of space manipulators with generalized jacobian matrix. IEEE Trans. Robot. Autom. 5, 303–314. 10.1109/70.34766

[B34] Valverde A. (2018). Dynamic Modeling and Control of Spacecraft Robotic Systems using Dual Quaternions. PhD thesis, Georgia Institute of Technology.

[B35] Virgili-Llop J. (2017). SPART: Spacecraft Robotics Toolkit. Available online at: https://github.com/NPS-SRL/SPART

[B36] Walker M. Weel L.-B. (1991). An adaptive control strategy for space based robot manipulators, in Proceedings of the IEEE International Conference on Robotics and Automation (Sacramento, CA).

[B37] Xu Y. Shum H.-Y. (1991). Dynamic control of a space robot system with no thrust jets controlled base. Technical Report CMU-RI-TR-91-33, Robotics Institute, Pittsburgh, PA.

[B38] Yoshida K. (1999). The SpaceDyn: a MATLAB toolbox for space and mobile robots, in Proceedings of the 1999 IEEE/RSJ International Conference on Intelligent Robots and Systems (Osaka), 1633–1638.

